# Circulating excitation–contraction coupling proteins and incident cardiovascular outcomes: associations and incremental predictive performance in the UK Biobank

**DOI:** 10.3389/fcvm.2026.1841253

**Published:** 2026-08-12

**Authors:** Dan Hu, Yang Gao, Jing Wang, Quan Guo, Zhuocheng Shi, Donghui Chen, Dongsheng Xia, Muwei Li, Xiaohu Wang, Bingchuan Geng

**Affiliations:** 1 Department of Cardiology, Fuwai Central China Cardiovascular Hospital, Central China Fuwai Hospital of Zhengzhou University, Zhengzhou, China; 2 Henan Provincial Clinical Research Center for Cardiovascular Disease, Fuwai Central China Cardiovascular Hospital, Zhengzhou, China

**Keywords:** excitation–contraction (E–C) coupling, heart failure, incident cardiovascular outcomes, plasma proteomics, risk prediction

## Abstract

**Introduction:**

Excitation–contraction coupling is central to cardiovascular physiology, but the prospective relevance of circulating proteins involved in this pathway remains uncertain.

**Methods:**

We evaluated a prespecified seven-protein excitation–contraction coupling panel (EC7: NEXN, SLMAP, ITPR1, CACNB1, HRC, RYR1, and CACNA1C) in the UK Biobank proteomics cohort in relation to incident stroke, heart failure, acute myocardial infarction, and angina recorded during the available observation period. Outcome-specific logistic-regression models estimated odds ratios per 1-standard-deviation higher relative protein level after adjustment for demographic and clinical factors, with false-discovery-rate correction across 28 primary tests. Predictive analyses used locked training/test partitions, screening of 13 candidate algorithms, nested cross-validation, frozen calibration and thresholds, and comparisons with clinical features and five established cardiovascular proteins.

**Results:**

Among 52,995 participants in the analysis source proteomics cohort, 39,691 had complete EC7 measurements. Higher ITPR1 (odds ratio, 1.088; 95% confidence interval, 1.033–1.146; q = 0.022) and HRC (odds ratio, 1.246; 95% confidence interval, 1.191–1.305; q < 0.001) were associated with incident heart failure; no other primary association survived correction. Logistic regression was selected for all outcomes. Adding EC7 proteins to clinical features did not improve locked-test predictive performance after multiplicity correction; for heart failure, changes were +0.0054 in ROC-AUC (q = 0.112) and +0.0084 in average precision (q = 0.217). Established comparator proteins consistently outperformed EC7 in biomarker-only comparisons.

**Discussion:**

Circulating excitation–contraction coupling proteins showed outcome-specific associations, particularly with heart failure, but limited incremental predictive performance. These findings support further mechanistic investigation and validation in independent cohorts.

## 1 Introduction

Cardiovascular diseases (CVDs) remain a major cause of morbidity and mortality and continue to motivate efforts to improve disease characterization and risk assessment beyond conventional clinical factors (1–3). Cardiac contraction depends on excitation–contraction (E–C) coupling, through which membrane depolarization triggers intracellular calcium release and converts an electrical signal into mechanical force (4–6). This process is coordinated by a structurally and functionally integrated network of ion channels, calcium-binding proteins, membrane-junction proteins, and cytoskeletal components that maintain calcium homeostasis and cardiomyocyte contractility (6, 7). Disruption of this network can impair calcium release, reuptake, and cellular architecture and has been implicated in heart failure, cardiac arrhythmias, ischemic injury, and adverse myocardial remodeling (8–12).

Several proteins involved in or closely linked to E–C coupling have established roles in maintaining cardiomyocyte structure and calcium handling. Nexilin, encoded by NEXN, contributes to junctional membrane complexes and cardiac T-tubule organization (13), whereas SLMAP isoforms influence cardiac gene expression and cellular function (14). Histidine-rich calcium-binding protein, encoded by HRC, modulates sarcoplasmic-reticulum calcium regulation and has been implicated in experimental heart failure and ischemia–reperfusion injury (15–17). Ryanodine receptors, voltage-dependent calcium-channel components, and inositol-trisphosphate receptors further contribute to intracellular calcium release and excitation–contraction signaling (4, 6, 8, 18, 19). Collectively, these observations support the biological relevance of circulating proteins related to the E–C coupling apparatus; however, tissue-level mechanistic importance does not necessarily imply that their plasma levels are informative at the population level.

Large-scale plasma proteomics has enabled systematic investigation of circulating proteins associated with incident myocardial infarction, ischemic stroke, heart failure, and other cardiovascular outcomes (20–22). Previous studies have shown that broad proteomic panels can identify cardiovascular risk signals and may provide modest improvements in event prediction (20, 21). Nevertheless, most such studies have evaluated agnostic or high-dimensional protein panels rather than a prespecified pathway-focused set. It therefore remains uncertain whether circulating proteins specifically related to E–C coupling show associations with distinct incident cardiovascular outcomes and whether they provide incremental predictive information beyond established clinical factors. An additional unresolved question is how their predictive performance compares with that of established circulating cardiovascular proteins.

We therefore investigated seven prespecified E–C coupling-related proteins—NEXN, SLMAP, ITPR1, CACNB1, HRC, RYR1, and CACNA1C—in the UK Biobank, a large prospective resource with linked phenotypic, proteomic, and health-record data (23, 24). We evaluated their outcome-specific associations with incident stroke, heart failure, acute myocardial infarction, and angina after adjustment for demographic and clinical factors and correction for multiple testing. We further assessed their incremental predictive performance using prespecified training procedures and locked internal test sets, and compared EC7-based models with clinical models and models incorporating five established cardiovascular comparator proteins. We hypothesized that selected circulating E–C coupling proteins would show outcome-specific associations with incident cardiovascular outcomes and evaluated whether they provided incremental predictive value beyond conventional clinical factors.

## 2 Materials and methods

### 2.1 Data source and study population

The UK Biobank is a prospective population-based cohort comprising approximately 500,000 participants aged 40–69 years who were recruited across England, Scotland, and Wales between 2006 and 2010 (23). At baseline, participants completed questionnaires and physical assessments and provided biological samples, with subsequent linkage to longitudinal health records (23, 24). The analysis source proteomics cohort comprised 52,995 unique participants. The analysis used the available UKB-PPP proteomics source cohort and was not restricted to the randomly selected baseline subgroup. Participants were not excluded on the basis of UKB-PPP consortium-selection status; therefore, consortium-selected participants were retained when present in the analysis source cohort. Of these, 39,691 had complete measurements of the seven prespecified excitation–contraction coupling proteins (EC7). Because prevalent-disease exclusions and data completeness differed by outcome, separate analytic cohorts were constructed for stroke, heart failure, acute myocardial infarction, and angina rather than applying a single common sample to all analyses. Participants with a qualifying diagnosis recorded on or before baseline were excluded from the corresponding outcome-specific cohort.

### 2.2 Plasma proteomics and protein panels

Baseline blood samples were collected, processed, aliquoted, and stored according to standardized UK Biobank procedures (24). Plasma protein measurements were generated within the UK Biobank Pharma Proteomics Project using the antibody-based Olink Explore 3072 proximity extension assay (25). The canonical analysis file contained 2,923 protein variables for the 52,995 participants in the analysis source proteomics cohort. Protein measurements were analyzed as relative protein levels without additional logarithmic transformation and were not interpreted as absolute plasma concentrations.

The primary pathway-focused panel comprised seven proteins involved in or related to excitation–contraction coupling: NEXN, SLMAP, ITPR1, CACNB1, HRC, RYR1, and CACNA1C. Participants with measurements available for all seven proteins constituted the complete EC7 cohort. The prespecified Comparator5 panel comprised NT-proBNP, GDF15, MMP12, HAVCR1, and TNFRSF10B. A secondary Common12 cohort required complete measurements of all seven EC7 proteins and all five Comparator5 proteins and was used for direct predictive-performance comparisons between the two protein panels.

### 2.3 Covariates and cardiovascular outcomes

The primary clinical covariates were selected before outcome modeling and comprised age, sex, body mass index, smoking status, systolic blood pressure, low-density lipoprotein cholesterol, hypertension, diabetes mellitus, blood-pressure medication use, cholesterol-lowering medication use, and income deprivation score. Age, body mass index, systolic blood pressure, low-density lipoprotein cholesterol, and income deprivation score were modeled as continuous variables. The income deprivation measure was the continuous UK Biobank income deprivation score for England (Field 26411); participants without this measure were excluded from the corresponding complete-case adjusted analyses. Smoking status was categorized as never, previous, or current smoking. Hypertension, diabetes mellitus, and medication-use variables were modeled as binary indicators. Ethnicity, employment status, alcohol consumption, diastolic blood pressure, and urinary albumin-to-creatinine ratio (uACR) were not included in the primary clinical adjustment model.

The four outcomes were incident ischemic or unspecified stroke, heart failure, acute myocardial infarction, and angina. Outcomes were derived from hospital inpatient ICD-10 diagnosis codes and their corresponding diagnosis dates in UK Biobank Fields 41270 and 41280 (23). Ischemic or unspecified stroke (hereafter referred to as stroke) was defined by ICD-10 codes beginning with I63 or I64; hemorrhagic stroke codes were not included. Heart failure was defined by codes beginning with I50, acute myocardial infarction by I21, and angina by I20. For follow-up construction, 31 October 2022 was used as the project-level proxy end date; this date was derived from the maximum observed hospital diagnosis-record date available in the project extract. For each outcome, the earliest qualifying diagnosis on or before baseline was classified as prevalent disease, whereas the earliest qualifying diagnosis after baseline and on or before the proxy end date was classified as an incident event. Participants without prevalent disease and without a qualifying incident event by the proxy end date were classified as eligible noncases. For time-to-event calculations, follow-up ended at the first qualifying event date for cases and at the earlier of death or the project-level proxy end date for noncases. Participants who died without a recorded qualifying event before death remained classified as noncases in the primary binary analyses. Death was not modeled as a competing event.

### 2.4 Descriptive and association analyses

Continuous baseline characteristics are presented as mean (standard deviation) or median [interquartile range], as appropriate, and categorical characteristics as counts with percentages. Because the primary objective of the baseline table was descriptive characterization rather than hypothesis testing, characteristics were summarized without relying on multiple univariable significance tests to define meaningful between-group differences. Missing clinical covariates were not imputed; adjusted association analyses used outcome-specific complete-case samples.

Associations between individual EC7 proteins and each cardiovascular outcome were evaluated using logistic regression because the prespecified primary endpoint was binary occurrence of the corresponding event during the available observation period. These models estimated the odds of event occurrence over the study-specific observation period and did not estimate a time-specific hazard or fixed-horizon absolute risk. Protein estimates were expressed as odds ratios with 95% confidence intervals per 1-standard-deviation higher relative protein level. For each outcome, the standard deviation was calculated in the full corresponding EC7 cohort before restriction to participants with complete clinical covariates. Model 1 adjusted for age and sex. The primary Model 2 additionally adjusted for body mass index, smoking status, systolic blood pressure, low-density lipoprotein cholesterol, hypertension, diabetes mellitus, blood-pressure medication use, cholesterol-lowering medication use, and income deprivation score. Model 3 included the Model 2 covariates and the five established cardiovascular comparator proteins and was fitted in the outcome-specific Common12 complete-case sample.

Heteroskedasticity-consistent type 3 (HC3) robust standard errors were used for all logistic-regression association models. The prespecified primary multiple-testing family comprised the 28 Model 2 protein–outcome tests—seven proteins across four outcomes—and was controlled using the Benjamini–Hochberg false discovery rate. Model 1 and Model 3 estimates were considered supportive and were not included in this primary false-discovery-rate family. All association estimates were interpreted as observational and noncausal.

### 2.5 Prediction-model development and evaluation

Prediction analyses were conducted using version-controlled scripts in a Linux environment with Python 3.11.15. The principal software packages included NumPy 2.4.6, pandas 2.3.3, SciPy 1.17.1, scikit-learn 1.9.0, imbalanced-learn 0.14.2, statsmodels 0.14.6, XGBoost 3.2.0, LightGBM 4.6.0, joblib 1.5.3, and Matplotlib 3.10.9. For each outcome, the prediction target was binary occurrence of the corresponding event during the available observation period. The models were not designed to estimate 5-year, 10-year, or other fixed-horizon absolute risks. Accordingly, ROC-AUC, average precision, Brier score, Brier skill score, log loss, and calibration measures refer to the study-specific binary endpoint over the available observation period. Separate outcome-specific partitions were created for stroke, heart failure, acute myocardial infarction, and angina. Each primary EC7 cohort was assigned to a stratified approximately 70:30 training–test partition using a fixed random seed of 42, with no participant overlap between the training and locked internal test sets. The Common12 cohorts inherited the corresponding primary-EC7 train/test assignments; participants were not redrawn. The locked test sets were not used for algorithm screening, hyperparameter tuning, calibration-method selection, classification-threshold selection, or feature selection.

Thirteen candidate algorithms were initially evaluated using five-fold cross-validation within the training data: random forest, bagging, k-nearest neighbors, XGBoost, decision tree, LightGBM, multilayer perceptron, support vector machine, gradient boosting, shallow neural network, AdaBoost, logistic regression, and naïve Bayes. Screening was based primarily on mean training-set cross-validated ROC-AUC, with average precision used as a secondary discrimination metric. The two highest-ranked candidates for each outcome proceeded to nested hyperparameter tuning using five outer folds and three inner folds, with 12 random-search iterations. Under the prespecified parsimonious selection rule, an alternative algorithm was selected over logistic regression only when its paired mean outer-fold ROC-AUC advantage exceeded one standard error of the paired differences.

After model selection, the selected algorithm was refitted using the full training sample. Logistic regression was selected for all four outcomes. The final logistic-regression models used the liblinear solver with a maximum of 5,000 iterations. Regularization was specified through the scikit-learn l1_ratio parameter, with l1_ratio=1 denoting pure L1 regularization and l1_ratio=0 denoting pure L2 regularization; outcome-specific C and l1_ratio values are reported in Supplementary Table S11. Calibration method and classification threshold were determined exclusively from cross-fitted training predictions and were frozen before the locked test sets were opened. Sigmoid calibration was used for stroke and heart failure, whereas isotonic calibration was used for acute myocardial infarction and angina. Within each training fold, missing predictor values were median-imputed and continuous predictors were standardized using parameters estimated exclusively from that fold. SMOTEENN was applied only to training folds and was never applied to validation-fold or locked-test observations.

Primary predictive comparisons evaluated the change in performance after adding EC7 proteins to the prespecified clinical feature set. Secondary analyses in the Common12 cohorts compared five feature sets: clinical factors alone, EC7 proteins alone, established comparator proteins alone, clinical factors plus EC7 proteins, and clinical factors plus established comparator proteins. Discrimination was assessed using ROC-AUC and average precision. Probabilistic performance and calibration were assessed using the Brier score, Brier skill score, log loss, calibration intercept, and calibration slope. Sensitivity, specificity, positive and negative predictive values, and balanced accuracy were calculated at the frozen outcome-specific thresholds. Where available, 95% confidence intervals were obtained from 2,000 successful bootstrap replicates. Model comparisons used paired bootstrap differences with false-discovery-rate adjustment within the prespecified comparison families. The locked test samples were internal holdout sets and were not interpreted as external or independent validation.

### 2.6 Sensitivity and exploratory analyses

Several revision-stage supportive and sensitivity analyses were conducted after the locked test evaluation and were not treated as independent validation or confirmatory analyses. First, an unsupervised first principal component was derived separately from the seven standardized EC7 proteins in each outcome-specific complete EC7 cohort. The resulting EC7 principal-component score was standardized and evaluated using the primary clinically adjusted logistic model; false-discovery-rate correction was applied across the four principal-component-outcome tests.

Second, exploratory Cox proportional-hazards models were fitted to evaluate time-to-event associations and were accompanied by proportional-hazards diagnostics. Follow-up time was calculated from baseline to the first qualifying event date for cases and to the earlier of death or the project-level proxy end date for noncases. As defined in Section 2.3, the proxy end date was 31 October 2022 and was derived from the maximum observed hospital diagnosis-record date available in the project extract. The Cox analyses were therefore considered exploratory, and associations showing evidence of violation of the proportional-hazards assumption were interpreted cautiously. Landmark analyses were additionally performed after excluding events occurring within the first 2 or 5 years after baseline to assess whether the primary association patterns were sensitive to early events.

Third, uACR was evaluated only in a complete-case sensitivity analysis because of its high level of missingness. uACR was calculated as urinary albumin divided by urinary creatinine and multiplied by 1,000, transformed using the natural logarithm, and standardized. For each protein-outcome pair, a clinical model without uACR and an otherwise identical model additionally adjusted for uACR were fitted in the same uACR-complete participants. These analyses were not interpreted as mediation analyses or as estimates of the proportion of an association explained by kidney function. Finally, characteristics, missingness patterns, and outcome rates were compared between participants with and without complete EC7 or Common12 measurements to evaluate potential selection related to protein-panel completeness.

## 3 Results

### 3.1 Study population and baseline characteristics

**Figure 1:**
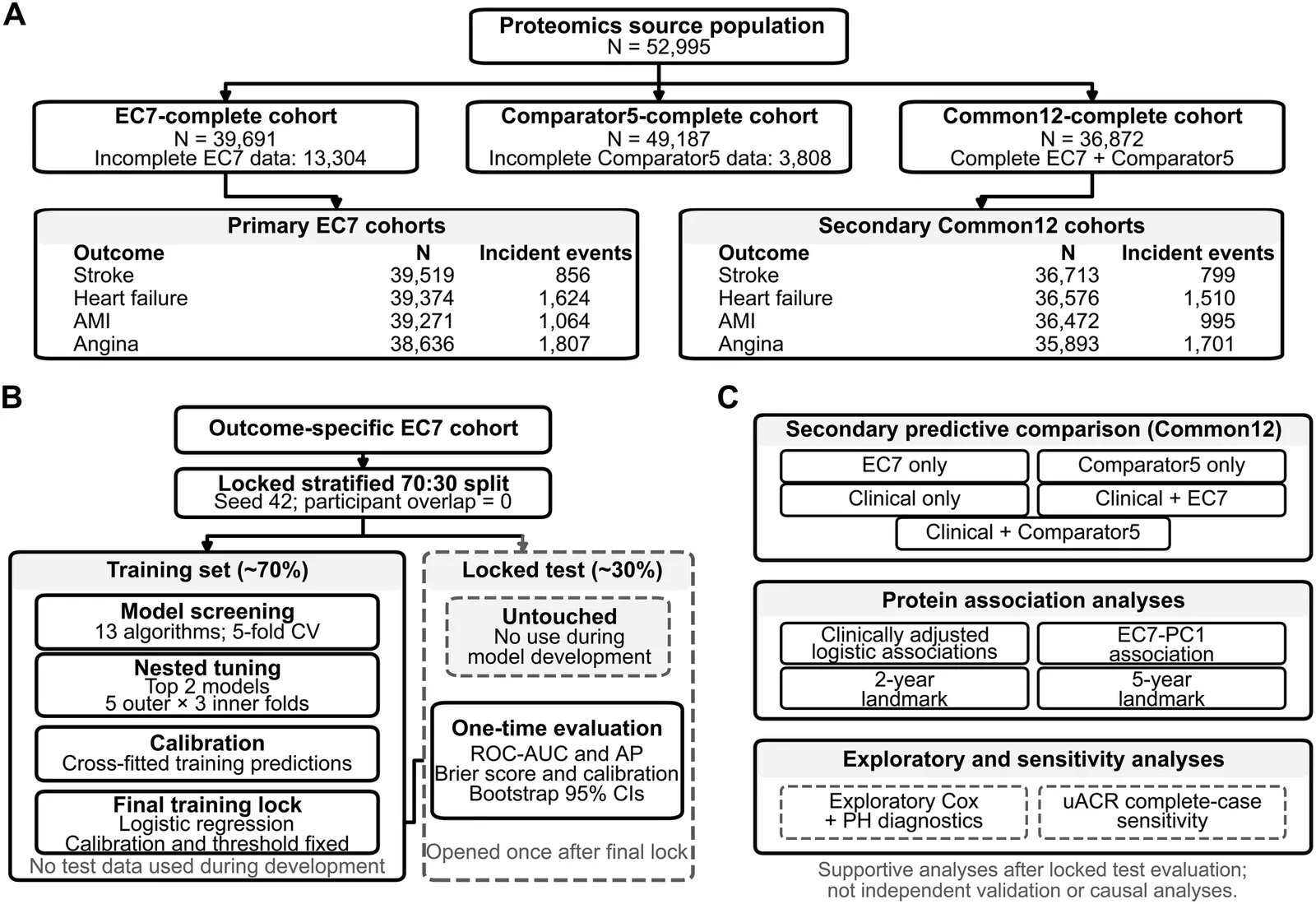
Study population and analysis workflow. **(A)** Derivation of the primary EC7 and secondary Common12 cohorts from the analysis source proteomics cohort of 52,995 participants. **(B)** Locked outcome-specific prediction workflow, including the stratified 70:30 training–test partition, algorithm screening, nested tuning, calibration, and final test-set evaluation. **(C)** Overview of the secondary predictive comparisons and supportive association and sensitivity analyses. EC7 denotes the seven excitation–contraction coupling proteins; Comparator5, the five established cardiovascular comparator proteins; and Common12, complete measurements of all seven EC7 and five Comparator5 proteins.

The study flow, outcome-specific analysis cohorts, locked prediction workflow, and supportive analyses are summarized in Figure 1. Among 52,995 participants in the analysis source proteomics cohort, 39,691 (74.9%) had complete measurements of all seven excitation–contraction coupling proteins (EC7), 49,187 (92.8%) had complete measurements of the five established comparator proteins, and 36,872 (69.6%) had complete measurements of all 12 proteins (Figure 1A; Supplementary Table S2). The operational definitions of the cardiovascular outcomes, EC7 proteins, and clinical covariates are provided in Supplementary Table S1. After outcome-specific exclusion of participants with prevalent disease or unavailable outcome information, the primary EC7 cohorts included 39,519 participants with 856 incident stroke events, 39,374 with 1,624 incident heart-failure events, 39,271 with 1,064 incident acute myocardial infarction events, and 38,636 with 1,807 incident angina events (Figure 1A; Table 1). Median follow-up was 13.59 years [interquartile range (IQR), 12.82–14.35] for stroke, 13.58 years (IQR, 12.79–14.35) for heart failure, 13.58 years (IQR, 12.80–14.35) for acute myocardial infarction, and 13.55 years (IQR, 12.75–14.34) for angina. In the corresponding outcome-specific cohorts, 3,855, 3,364, 3,776, and 3,510 participants, respectively, died without a recorded target event and remained classified as noncases in the primary binary analyses.

**Table 1:** Baseline characteristics and incident cardiovascular outcomes among participants with complete EC7 protein measurements (*N* = 39,691).

Characteristic	Available N	Value
Demographic and lifestyle characteristics		
Age, years	39,691	56.8 (8.2)
Male sex	39,691	18,212 (45.9)
Smoking status	39,499	
Never	39,499	21,416 (54.2)
Previous	39,499	13,817 (35.0)
Current	39,499	4,266 (10.8)
Clinical characteristics		
Body mass index, kg/m^2^	39,501	27.5 (4.8)
Systolic blood pressure, mmHg	39,654	137.8 (18.7)
LDL cholesterol, mmol/L	37,621	3.53 (0.88)
Hypertension	39,671	11,960 (30.1)
Diabetes mellitus	39,674	2,282 (5.8)
Blood-pressure medication	39,691	8,743 (22.0)
Cholesterol-lowering medication	39,163	7,339 (18.7)
Income deprivation score	33,967	0.119 (0.101)
Urinary albumin-to-creatinine ratio		
uACR available	39,691	12,434 (31.3)
uACR, mg/mmol	12,434	1.07 [0.66, 2.17]
Incident cardiovascular outcomes		
Incident stroke	39,519	856 (2.2)
Incident heart failure	39,374	1,624 (4.1)
Incident acute myocardial infarction	39,271	1,064 (2.7)
Incident angina	38,636	1,807 (4.7)

Values are presented as mean (standard deviation), median [interquartile range], or *n* (%), as appropriate. Percentages are based on participants with available data for the corresponding characteristic. The EC7 cohort was defined before outcome-specific exclusions; outcome rows therefore use the eligible denominators shown in the Available N column.

Among participants with complete EC7 measurements, the mean age was 56.8 years (SD, 8.2), 45.9% were male, the mean body mass index was 27.5 kg/m^2^, and the mean systolic blood pressure was 137.8 mmHg. Hypertension and diabetes mellitus were present in 30.1% and 5.8% of participants, respectively. Urinary albumin-to-creatinine ratio (uACR) was available for 12,434 participants (31.3%), with a median value of 1.07 mg/mmol (interquartile range, 0.66–2.17) (Table 1).

In the descriptive selection audit, all measured baseline differences between participants with complete and incomplete EC7 measurements, and between those with complete and incomplete Common12 measurements, were below the prespecified absolute standardized mean difference threshold of 0.10 (Supplementary Figure S1; Supplementary Table S10). The largest observed differences involved available uACR measurements, with absolute standardized mean differences of 0.086 for EC7 completeness and 0.087 for Common12 completeness. Unadjusted outcome-rate differences between complete- and incomplete-panel groups were generally small; the largest differences were observed for heart failure, for which the event rate was 0.62 percentage points lower in the EC7-complete group and 0.50 percentage points lower in the Common12-complete group (Supplementary Table S10).

### 3.2 Associations of EC7 proteins with incident cardiovascular outcomes

**Figure 2:**
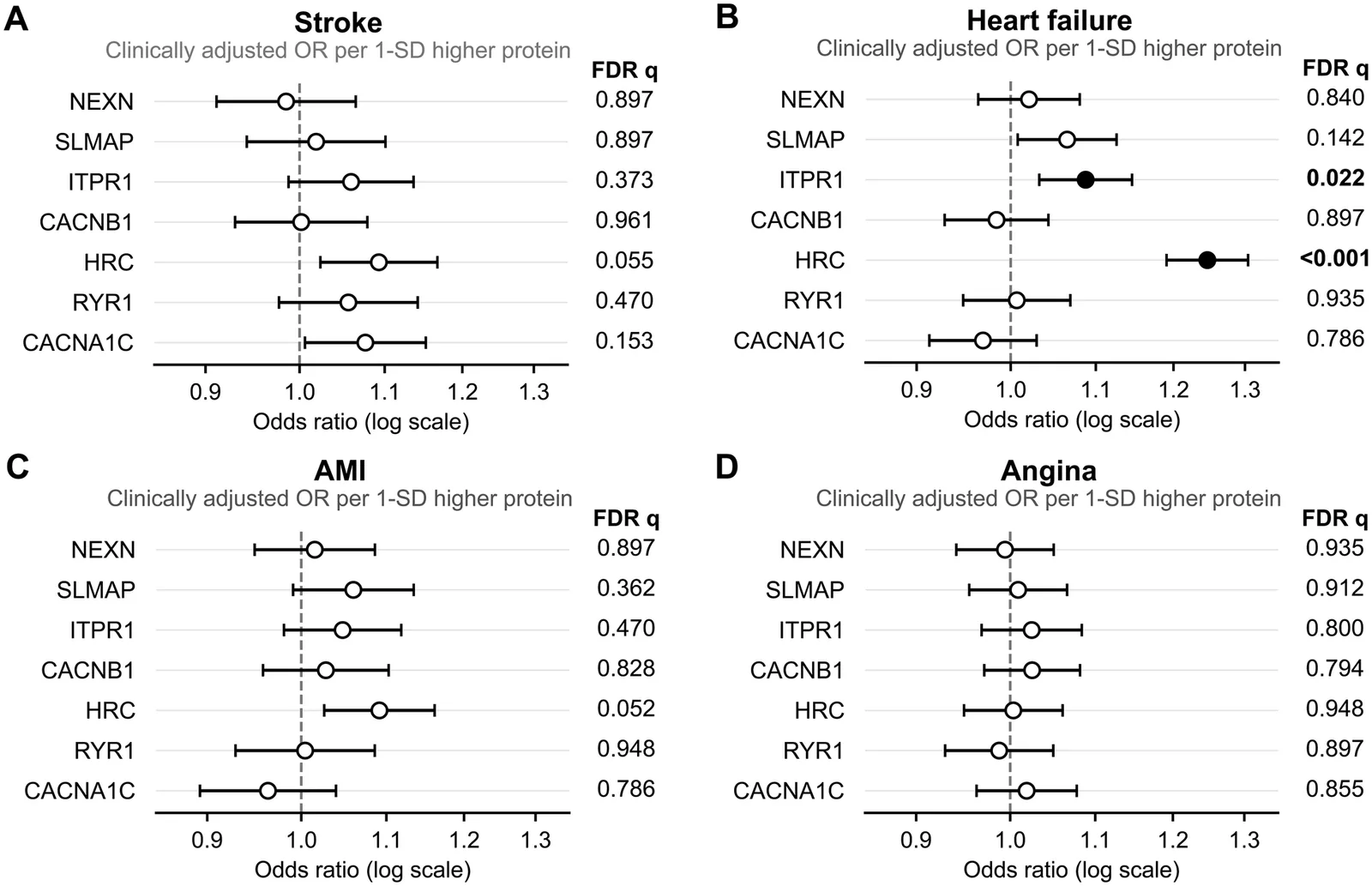
Clinically adjusted associations of EC7 proteins with incident cardiovascular outcomes. **(A)** Stroke. **(B)** Heart failure. **(C)** Acute myocardial infarction. **(D)** Angina. Odds ratios are reported per 1-standard-deviation higher relative protein level from the primary clinically adjusted logistic-regression model. Horizontal lines indicate 95% confidence intervals, and the dashed vertical line denotes an odds ratio of 1. Filled circles indicate Benjamini–Hochberg false-discovery-rate q < 0.05 across the 28 primary protein–outcome tests.

The primary clinically adjusted association analyses included 31,395 participants with 655 incident stroke events, 31,272 with 1,284 heart-failure events, 31,193 with 842 acute myocardial infarction events, and 30,674 with 1,446 angina events. After Benjamini–Hochberg correction across the 28 prespecified protein–outcome tests, two associations met the false-discovery-rate threshold, both for incident heart failure (Figure 2; Table 2). Each 1-SD higher ITPR1 level was associated with an odds ratio (OR) of 1.088 for heart failure [95% confidence interval (CI), 1.033–1.146; *P* = 0.002; q = 0.022], while each 1-SD higher HRC level was associated with an OR of 1.246 (95% CI, 1.191–1.305; *P* < 0.001; q < 0.001). No other primary protein–outcome association met q < 0.05.

**Table 2:** Adjusted associations of EC7 proteins with incident cardiovascular outcomes.

Outcome	Protein	N	Cases	Adjusted OR (95% CI)	*P* value	FDR-adjusted q value
Stroke	NEXN	31,395	655	0.985 (0.911−1.065)	0.704	0.897
Stroke	SLMAP	31,395	655	1.019 (0.943–1.101)	0.640	0.897
Stroke	ITPR1	31,395	655	1.059 (0.988–1.136)	0.106	0.373
Stroke	CACNB1	31,395	655	1.002 (0.930–1.079)	0.961	0.961
Stroke	HRC	31,395	655	1.093 (1.024–1.167)	0.008	0.055
Stroke	RYR1	31,395	655	1.056 (0.977–1.141)	0.168	0.470
Stroke	CACNA1C	31,395	655	1.076 (1.006–1.152)	0.033	0.153
Heart failure	NEXN	31,272	1,284	1.021 (0.964–1.080)	0.480	0.840
Heart failure	SLMAP	31,272	1,284	1.065 (1.008–1.126)	0.025	0.142
Heart failure	ITPR1	31,272	1,284	1.088 (1.033–1.146)	0.002	0.022
Heart failure	CACNB1	31,272	1,284	0.984 (0.929–1.043)	0.592	0.897
Heart failure	HRC	31,272	1,284	1.246 (1.191–1.305)	<0.001	<0.001
Heart failure	RYR1	31,272	1,284	1.007 (0.948–1.069)	0.825	0.935
Heart failure	CACNA1C	31,272	1,284	0.969 (0.913–1.029)	0.311	0.786
Acute myocardial infarction	NEXN	31,193	842	1.015 (0.949–1.086)	0.660	0.897
Acute myocardial infarction	SLMAP	31,193	842	1.060 (0.991–1.134)	0.091	0.362
Acute myocardial infarction	ITPR1	31,193	842	1.047 (0.981–1.118)	0.167	0.470
Acute myocardial infarction	CACNB1	31,193	842	1.028 (0.958–1.103)	0.444	0.828
Acute myocardial infarction	HRC	31,193	842	1.091 (1.026–1.161)	0.006	0.052
Acute myocardial infarction	RYR1	31,193	842	1.004 (0.929–1.086)	0.914	0.948
Acute myocardial infarction	CACNA1C	31,193	842	0.963 (0.893–1.040)	0.337	0.786
Angina	NEXN	30,674	1,446	0.994 (0.941–1.050)	0.835	0.935
Angina	SLMAP	30,674	1,446	1.009 (0.955–1.066)	0.749	0.912
Angina	ITPR1	30,674	1,446	1.024 (0.968–1.084)	0.400	0.800
Angina	CACNB1	30,674	1,446	1.025 (0.971–1.081)	0.369	0.794
Angina	HRC	30,674	1,446	1.004 (0.950–1.061)	0.900	0.948
Angina	RYR1	30,674	1,446	0.988 (0.930–1.050)	0.691	0.897
Angina	CACNA1C	30,674	1,446	1.019 (0.963–1.077)	0.519	0.855

Odds ratios and 95% confidence intervals are reported per 1-standard-deviation higher relative protein level; the standard deviation was calculated in the full corresponding outcome-specific EC7 cohort before covariate complete-case restriction. Models adjusted for age, sex, body mass index, smoking status, systolic blood pressure, low-density lipoprotein cholesterol, hypertension, diabetes mellitus, blood-pressure medication, cholesterol-lowering medication, and income deprivation score, with HC3 robust standard errors. Benjamini–Hochberg false-discovery-rate correction was applied across the 28 primary protein–outcome tests; bold q values indicate q < 0.05.

The nominal associations of HRC with stroke (OR, 1.093; 95% CI, 1.024–1.167; q = 0.055) and acute myocardial infarction (OR, 1.091; 95% CI, 1.026–1.161; q = 0.052) did not meet the prespecified false-discovery-rate threshold (Figure 2; Table 2). Complete estimates from the age- and sex-adjusted model, the primary clinically adjusted model, and the supportive model additionally adjusted for the five comparator proteins are reported in Supplementary Table S5. In the smaller Common12 complete-case samples, additional comparator-protein adjustment attenuated the heart-failure estimates for ITPR1 (OR, 1.051; 95% CI, 0.990–1.116; *P* = 0.103) and HRC (OR, 1.044; 95% CI, 0.978–1.114; *P* = 0.196); these supportive estimates were not included in the primary multiple-testing family (Supplementary Table S5).

### 3.3 Prediction-model development and locked-test performance

For each outcome, participants were assigned to an approximately 70:30 stratified training–test partition, with no overlap between the training and locked internal test sets (Figure 1B; Supplementary Table S12). The Common12 cohorts inherited the corresponding primary-EC7 train/test assignments, and no Common12 participant was redrawn. All algorithm screening, hyperparameter tuning, calibration-method selection, and classification-threshold selection were completed without using locked test-set observations (Figures 1B,C; Supplementary Table S12).

During training-set screening, logistic regression had the highest mean five-fold cross-validated ROC-AUC for all four outcomes: 0.575 for stroke, 0.591 for heart failure, 0.563 for acute myocardial infarction, and 0.546 for angina (Supplementary Figure S2; Supplementary Table S11). The two highest-ranked algorithms for each outcome underwent nested cross-validation. None of the alternative algorithms exceeded logistic regression by more than one standard error of the paired outer-fold ROC-AUC differences; therefore, logistic regression was selected for all four outcomes under the prespecified parsimony rule (Supplementary Table S11).

The primary EC7 locked test sets included 11,856 participants with 257 stroke events, 11,813 with 487 heart-failure events, 11,782 with 319 acute myocardial infarction events, and 11,591 with 542 angina events (Supplementary Table S12). EC7-only logistic-regression models showed modest discrimination, with locked-test ROC-AUCs of 0.549 (95% CI, 0.512–0.585) for stroke, 0.606 (95% CI, 0.579–0.632) for heart failure, 0.520 (95% CI, 0.489–0.552) for acute myocardial infarction, and 0.539 (95% CI, 0.515–0.563) for angina. Corresponding average precision values were 0.026, 0.071, 0.029, and 0.054, respectively (Supplementary Figure S3; Supplementary Table S3).

Sigmoid calibration was used for stroke and heart failure, whereas isotonic calibration was used for acute myocardial infarction and angina; all calibration procedures and classification thresholds were frozen before the locked test sets were opened (Supplementary Figure S4; Supplementary Tables S3,S12). Calibrated Brier scores were 0.0212 for stroke, 0.0393 for heart failure, 0.0264 for acute myocardial infarction, and 0.0446 for angina. Brier skill scores were small—0.0005, 0.0064, −0.0014, and −0.0001, respectively—indicating little improvement over prediction based only on the outcome-specific event rate. Calibration plots and intercept–slope estimates indicated residual calibration departures across outcomes, with the largest departures observed for acute myocardial infarction and angina (Supplementary Figure S4; Supplementary Table S3).

**Figure 3:**
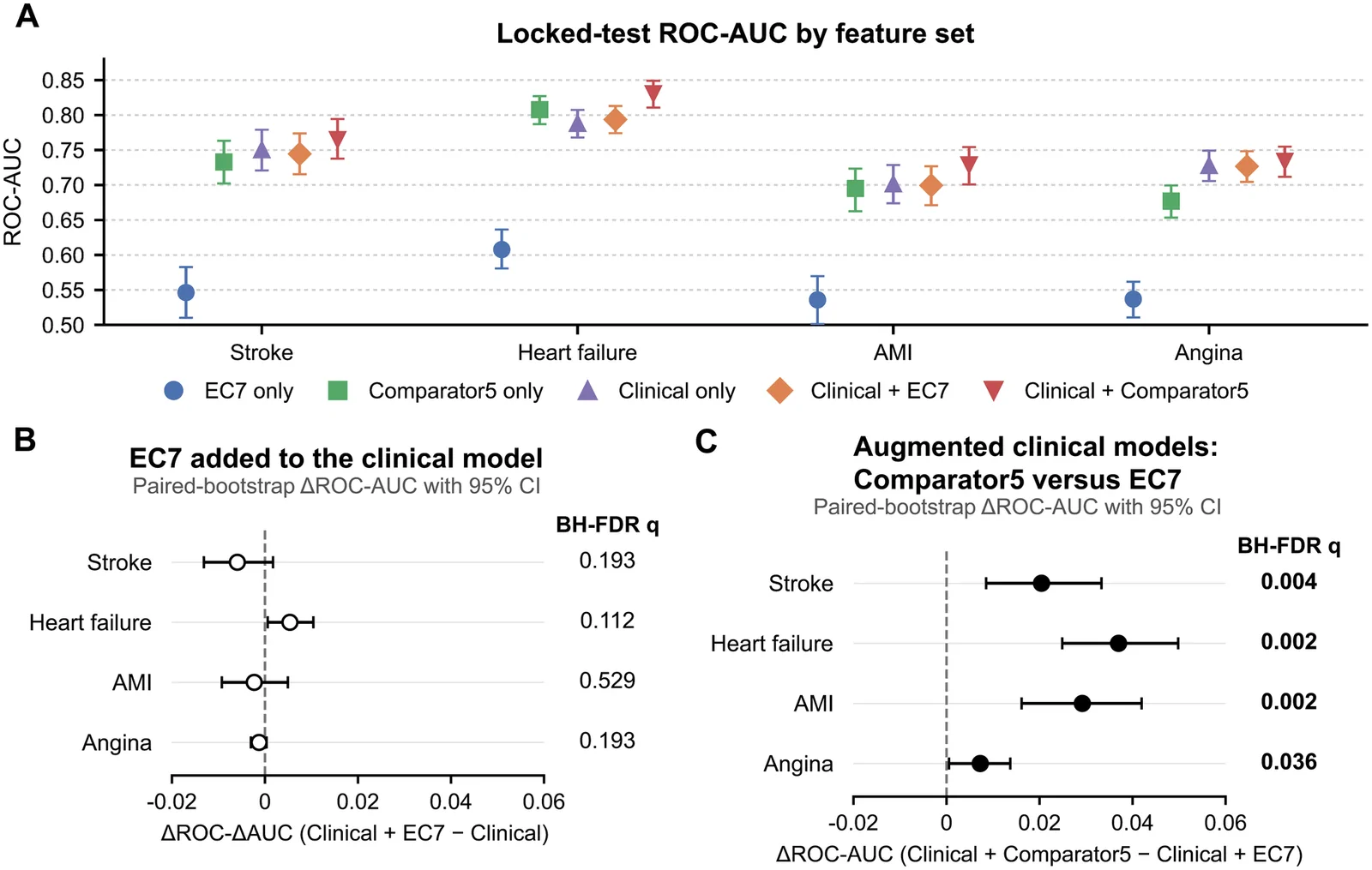
Locked-test discrimination of EC7 and established comparator proteins. **(A)** ROC-AUCs for the five prespecified feature sets in the outcome-specific Common12 locked test sets. **(B)** Paired change in ROC-AUC after adding EC7 proteins to the clinical model. **(C)** Paired difference in ROC-AUC between the clinical-plus-Comparator5 and clinical-plus-EC7 models. Error bars indicate bootstrap 95% confidence intervals; positive differences favor the first-named model. Filled circles indicate Benjamini–Hochberg false-discovery-rate q < 0.05, and dashed vertical lines denote no difference.

In the Common12 locked test sets, adding EC7 proteins to the clinical model did not improve ROC-AUC after correction for the four outcome-specific comparisons (Figure 3B; Table 3). The paired changes in ROC-AUC were −0.0059 for stroke (95% CI, −0.0131 to 0.0017; q = 0.193), +0.0054 for heart failure (95% CI, 0.0006–0.0104; q = 0.112), −0.0023 for acute myocardial infarction (95% CI, −0.0093 to 0.0049; q = 0.529), and −0.0013 for angina (95% CI, −0.0030 to 0.0003; q = 0.193). None of the primary incremental comparisons based on ROC-AUC, average precision, Brier score, Brier skill score, or log loss met the corresponding false-discovery-rate threshold (Supplementary Table S4).

**Table 3:** Incremental discrimination of EC7 proteins beyond clinical predictors in the locked test set.

Outcome	Test N	Cases	Clinical ROC-AUC (95% CI)	Clinical + EC7 ROC-AUC (95% CI)	*Δ*ROC-AUC (95% CI)	Bootstrap *P* value	FDR-adjusted q value
Stroke	10,986	241	0.750 (0.721 to 0.779)	0.745 (0.715 to 0.774)	−0.0059 (−0.0131 to +0.0017)	0.145	0.193
Heart failure	10,990	457	0.788 (0.768 to 0.807)	0.794 (0.774 to 0.813)	+0.0054 (+0.0006 to +0.0104)	0.028	0.112
Acute myocardial infarction	10,935	295	0.702 (0.674 to 0.728)	0.699 (0.671 to 0.727)	−0.0023 (−0.0093 to +0.0049)	0.529	0.529
Angina	10,802	509	0.728 (0.706 to 0.749)	0.727 (0.705 to 0.748)	−0.0013 (−0.0030 to +0.0003)	0.134	0.193

ROC-AUCs were evaluated from raw predicted probabilities in the outcome-specific Common12 locked test sets. *Δ*ROC-AUC was calculated as Clinical + EC7 minus Clinical; positive values favor the addition of EC7 proteins. Confidence intervals and paired *P* values were estimated using 2,000 bootstrap replicates, and Benjamini–Hochberg false-discovery-rate correction was applied across the four outcome-specific comparisons. No model-development decisions were made using locked test data.

Established comparator proteins consistently provided greater discrimination than EC7 proteins in the locked Common12 test sets (Figures 3A,3C; Supplementary Table S4). Compared with clinical-plus-EC7 models, clinical-plus-comparator-protein models had higher ROC-AUCs for stroke (difference, +0.0204; 95% CI, 0.0085–0.0333; q = 0.004), heart failure (+0.0370; 95% CI, 0.0249–0.0498; q = 0.002), acute myocardial infarction (+0.0292; 95% CI, 0.0161–0.0419; q = 0.002), and angina (+0.0072; 95% CI, 0.0005–0.0137; q = 0.036). Comparator proteins also outperformed EC7 proteins in all four biomarker-only ROC-AUC and average-precision comparisons (all q < 0.001) (Figure 3; Supplementary Table S4).

### 3.4 Supportive and sensitivity analyses

The first principal component of the seven EC7 proteins explained 28.81%–28.86% of their total variance across the four outcome-specific cohorts (Supplementary Table S6). A higher standardized EC7 first-principal-component score was associated with incident heart failure (OR per 1-SD increase, 1.102; 95% CI, 1.043–1.165; q = 0.002), but not with stroke, acute myocardial infarction, or angina after correction across the four principal-component analyses (Supplementary Figure S5A; Supplementary Table S6).

In landmark analyses, HRC remained associated with heart failure after excluding events occurring within the first 2 years (OR, 1.222; 95% CI, 1.165–1.281; q < 0.001) and the first 5 years (OR, 1.214; 95% CI, 1.154–1.277; q < 0.001) (Supplementary Figure S5B–I; Supplementary Table S7). At the 5-year landmark, CACNA1C was also associated with stroke (OR, 1.115; 95% CI, 1.038–1.197; q = 0.038). No other landmark association met the corresponding false-discovery-rate threshold (Supplementary Figure S5; Supplementary Table S7).

In exploratory Cox analyses, HRC was associated with stroke [hazard ratio (HR), 1.108; 95% CI, 1.038–1.182; q = 0.015], heart failure (HR, 1.224; 95% CI, 1.180–1.269; q < 0.001), and acute myocardial infarction (HR, 1.103; 95% CI, 1.040–1.170; q = 0.015). SLMAP and ITPR1 were also associated with heart failure (both q = 0.015) (Supplementary Figure S6; Supplementary Table S8). However, the proportional-hazards diagnostic for the heart failure–HRC model remained significant after multiple-testing correction (q = 0.001), indicating that its single summary HR should be interpreted cautiously. These analyses were additionally limited by the use of 31 October 2022 as a project-level proxy end date derived from the maximum observed hospital diagnosis-record date in the project extract, rather than from a separately documented follow-up schedule (Supplementary Figure S6; Supplementary Table S8).

Because approximately 69% of participants in the primary clinically complete samples lacked uACR measurements, the uACR sensitivity analyses were restricted to 9,445–9,729 participants, depending on outcome (Supplementary Figure S7; Supplementary Table S9). After additional adjustment for standardized log-transformed uACR, HRC remained associated with heart failure (OR, 1.213; 95% CI, 1.129–1.303; q < 0.001), whereas the corresponding ITPR1 estimate was attenuated to 1.011 (95% CI, 0.930–1.100; q = 0.922). No other uACR-adjusted association met the false-discovery-rate threshold. These changes were descriptive within a restricted complete-case population and were not interpreted as evidence of mediation or causality (Supplementary Figure S7; Supplementary Table S9).

## 4 Discussion

In this large prospective UK Biobank study, we evaluated the associations and predictive relevance of seven circulating proteins involved in or related to excitation–contraction coupling across four incident cardiovascular outcomes. Three principal findings emerged. First, after adjustment for demographic and clinical factors and false-discovery-rate correction across 28 primary tests, higher circulating ITPR1 and HRC levels were associated with incident heart failure, whereas no other primary protein–outcome association met the prespecified false-discovery-rate threshold (Figure 2; Table 2). Second, a supportive unsupervised EC7 first-principal-component score was also associated with heart failure, and the association between HRC and heart failure persisted in both the 2-year and 5-year landmark analyses (Supplementary Figure S5; Supplementary Tables S6,S7). Third, EC7 proteins showed limited predictive performance when evaluated in locked internal test sets. Adding EC7 proteins to clinical predictors did not improve discrimination or other predictive-performance measures after multiplicity correction, while the established comparator-protein panel generally outperformed EC7 in both biomarker-only and clinically augmented comparisons (Figure 3; Table 3 and Supplementary Table S4). These findings distinguish pathway-related epidemiological associations from clinically meaningful incremental prediction.

The strongest primary association was observed between HRC and incident heart failure. HRC is a sarcoplasmic-reticulum calcium-binding protein that participates in the regulation of calcium storage and release, and experimental studies have linked altered HRC expression or function to impaired calcium handling, ventricular dysfunction, ischemia–reperfusion injury, and cardiomyopathic phenotypes (15–17) More broadly, abnormal intracellular calcium cycling is a central feature of adverse myocardial remodeling and heart failure (5, 6, 8–11) In the present study, each 1-SD higher circulating HRC level was associated with 24.6% higher odds of incident heart failure in the primary clinically adjusted analysis, and the direction and magnitude of this association remained broadly consistent in the landmark analyses (Figure 2; Supplementary Figure S5; Table 2 and Supplementary Table S7). HRC also remained associated with heart failure in the uACR-complete sample after additional adjustment for uACR, although this analysis was restricted to approximately 31% of the clinically complete population (Supplementary Figure S7; Supplementary Table S9). Taken together, these results support HRC as a candidate circulating marker of processes related to heart-failure development, but they do not establish that circulating HRC directly participates in disease initiation or that modification of HRC would alter clinical risk.

ITPR1 was the second EC7 protein associated with incident heart failure after correction for multiple testing. Inositol 1,4,5-trisphosphate receptors regulate intracellular calcium release and contribute to calcium-dependent signaling in cardiomyocytes and vascular cells (4, 8, 11, 19). However, the ITPR1 association was less consistent across supportive analyses than the HRC association. The primary clinically adjusted odds ratio was 1.088 per 1-SD higher ITPR1 level, but the estimate was attenuated after additional adjustment for the five comparator proteins and in the restricted uACR-complete population (Table 2; Supplementary Tables S5,S9). ITPR1 also did not meet the false-discovery-rate threshold in either landmark analysis. These differences may reflect confounding, shared information with established cardiovascular proteins, reduced precision in smaller complete-case samples, or variation related to participant selection. They therefore argue against interpreting circulating ITPR1 as an independently established heart-failure biomarker at this stage.

The concentration of the most robust associations in heart failure is biologically plausible because excitation–contraction coupling and calcium cycling are directly involved in myocardial contraction, relaxation, energetic demand, and ventricular remodeling (5, 6, 8–12) By contrast, stroke, acute myocardial infarction, and angina encompass heterogeneous vascular, thrombotic, atherosclerotic, and ischemic mechanisms that may not be adequately represented by a single baseline measurement of a small calcium-handling protein panel (26–28) Although HRC showed nominal associations with stroke and acute myocardial infarction in the primary logistic analyses, neither met the prespecified false-discovery-rate threshold (Figure 2; Table 2). A 5-year landmark association between CACNA1C and stroke and several significant exploratory Cox associations should be considered supportive rather than confirmatory because they arose from separate multiple-testing families and were not uniformly reproduced across analyses (Supplementary Figures S5,S6; Supplementary Tables S7,S8).

The supportive EC7 principal-component analysis provided complementary evidence for a pathway-level association with heart failure. The first principal component explained approximately 29% of the variation in the seven proteins and was associated with incident heart failure after correction across four outcomes, but not with stroke, acute myocardial infarction, or angina (Supplementary Figure S5; Supplementary Table S6). Because NEXN and SLMAP had the largest positive loadings, this component primarily represented their shared variation rather than a heart-failure-specific combination of the strongest individual proteins. The analysis was unsupervised and did not use outcome labels, but it was derived from the same participants and proteins as the primary analyses. It should therefore be viewed as supportive pathway-level evidence rather than independent replication or proof of a latent biological mechanism. NEXN and SLMAP have established structural roles in junctional membrane organization, T-tubule integrity, and cardiac cellular function (13, 14), but their circulating levels were not independently associated with heart failure after correction in the primary analysis.

A central finding of the prediction analyses was that statistical association did not translate into substantial incremental predictive performance. Logistic regression was selected for all four outcomes after training-set screening and nested cross-validation; none of the alternative algorithms satisfied the prespecified criterion for replacing the more parsimonious model (Supplementary Figure S2; Supplementary Table S11). EC7-only models had modest locked-test discrimination, with ROC-AUCs ranging from 0.520 for acute myocardial infarction to 0.606 for heart failure, and average precision values remained close to the low underlying event rates (Supplementary Figure S3; Supplementary Table S3). Calibration and Brier skill results similarly indicated limited improvement over event-rate-only prediction, particularly for stroke, acute myocardial infarction, and angina (Supplementary Figure S4; Supplementary Table S3). These results do not support interpreting EC7 proteins as strong predictors of cardiovascular disease or inferring that complex nonlinear algorithms provide high discrimination in this setting. Previous studies have evaluated a range of individual and ensemble machine-learning approaches for stroke and cardiovascular disease prediction using conventional clinical datasets (29, 30). These studies illustrate the broad methodological interest in machine-learning-based cardiovascular risk assessment, although their data sources, target populations, and prediction objectives differ substantially from those of the present pathway-focused plasma-proteomic analysis.

When added to clinical predictors, EC7 proteins produced a small nominal increase in heart-failure ROC-AUC but no improvement remained significant after correction for the four outcome comparisons. Changes in ROC-AUC were negative or close to zero for stroke, acute myocardial infarction, and angina (Figure 3; Table 3). Similar conclusions were obtained for average precision, Brier score, Brier skill score, and log loss (Supplementary Table S4). In contrast, the established comparator-protein panel improved discrimination relative to EC7 for all four outcomes, whether the protein panels were evaluated alone or together with clinical predictors (Figure 3; Supplementary Table S4). For example, in heart failure, the established comparator-only model had a ROC-AUC of 0.808 compared with 0.608 for EC7 alone, while the clinical-plus-comparator model had a ROC-AUC of 0.831 compared with 0.794 for the clinical-plus-EC7 model. These comparisons indicate that pathway specificity and mechanistic interest do not necessarily confer greater prognostic information than proteins that capture broader cardiovascular stress, inflammation, renal dysfunction, myocardial strain, or tissue injury.

Our prediction findings are broadly consistent with previous large-scale proteomic studies showing that circulating proteins can contribute to cardiovascular risk prediction but that improvements beyond clinical variables are often modest and depend on the population, outcome definition, number of proteins, and validation design (20–22) Royer et al. reported modest improvements in major cardiovascular-event prediction using panels derived from approximately 2,900 UK Biobank proteins (21). More recently, Luo et al. developed a multitask deep-learning framework using 2,920 proteins and 168 metabolites from the UK Biobank to derive disease-specific omics risk scores across six cardiovascular outcomes, illustrating the predictive potential of broad, data-driven multiomic panels (31). Xiang et al. developed a multivariable protein model in survivors of myocardial infarction using a substantially larger initial protein set and a clinically different target population (32). Direct numerical comparisons with the present study would be inappropriate because our analysis used seven prespecified pathway-related proteins, four distinct incident outcomes, outcome-specific locked internal test sets, and different event prevalences. Nevertheless, the contrast illustrates the difficulty of obtaining clinically meaningful prediction from a small biologically selected panel, even when individual proteins show epidemiological associations.

The exploratory Cox analyses showed FDR-significant associations of HRC with stroke, heart failure, and acute myocardial infarction and of SLMAP and ITPR1 with heart failure (Supplementary Figure S6; Supplementary Table S8). However, these findings require particular caution. For the Cox analyses, follow-up was constructed using 31 October 2022 as a project-level proxy end date derived from the maximum observed hospital diagnosis-record date in the project extract. The proportional-hazards assumption was also not supported for the heart failure–HRC model after correction for multiple testing, meaning that a single constant hazard ratio may inadequately summarize that association over time. The primary logistic analyses addressed binary event occurrence during the available observation period and did not explicitly account for variation in follow-up duration or the competing risk of death. The Cox analyses should therefore be regarded as exploratory evidence concerning the direction of time-to-event associations rather than as definitive estimates for a prespecified follow-up horizon.

This study has several strengths. It used a large prospective cohort, prespecified a biologically focused EC7 panel, evaluated four outcome-specific cohorts, applied robust standard errors and correction across a clearly defined primary multiple-testing family, and distinguished association analyses from prediction analyses. The prediction workflow separated model development from final evaluation through locked stratified training–test partitions, nested tuning, training-derived calibration, and thresholds fixed before test-set opening (Figure 1; Supplementary Table S12). Direct comparisons with an established cardiovascular protein panel were conducted within the same Common12 participants and locked test sets, reducing differences attributable to sample composition. Multiple supportive analyses—including principal-component, landmark, Cox, uACR, and protein-completeness analyses—were used to examine the consistency and limitations of the main findings rather than being treated as independent validation.

Several limitations should also be considered. First, the study was observational, and residual confounding, reverse causation, and subclinical disease cannot be excluded. The reported odds ratios represent associations and should not be interpreted as causal effects or risk ratios. Second, UK Biobank participants are not fully representative of the general population. Moreover, the UKB-PPP proteomics source cohort included participants selected through both random and non-random sampling mechanisms, including consortium-selected participants. Consequently, the analysis source cohort should not be considered representative of either the complete UK Biobank cohort or the general population. Completeness of the EC7 and Common12 protein panels and the complete-case restriction for clinical covariates may have introduced additional selection. In addition, use of the England-specific income deprivation score (UK Biobank Field 26411), together with complete-case exclusion of participants without this measure, may have introduced additional geographic selection. Although measured baseline differences according to EC7 and Common12 completeness were small, selection related to unmeasured characteristics and residual selection bias cannot be excluded (Supplementary Figure S1; Supplementary Table S10) (23). Third, proteins were measured once at baseline as relative circulating levels. Plasma measurements integrate expression, tissue release, degradation, distribution, and clearance and may not reflect protein abundance or function within cardiomyocytes, vascular smooth muscle, or other specific tissues. Therefore, mechanistic interpretations based on intracellular functions should remain cautious.

Fourth, outcomes were identified from hospital ICD-10 records. These definitions do not capture all clinically relevant phenotypes or disease subtypes and cannot reliably distinguish obstructive epicardial coronary disease from microvascular angina, different heart-failure phenotypes, or specific ischemic stroke mechanisms. Moreover, the stroke outcome was restricted to ICD-10 codes I63 and I64 and therefore represented ischemic or unspecified stroke rather than an all-stroke endpoint; hemorrhagic stroke events were not included. Accordingly, the present findings should not be generalized to all stroke subtypes. In addition, the present data do not support claims that EC7 proteins are direct markers of coronary microvascular dysfunction, vascular-wall stability, or heart failure with preserved ejection fraction. Fifth, the primary adjusted analyses used complete clinical-covariate data rather than imputation. uACR was unavailable for approximately 69% of otherwise eligible clinically complete participants and could only be evaluated in a substantially restricted complete-case sensitivity analysis (Supplementary Figure S7; Supplementary Table S9). Changes in coefficients after uACR adjustment should not be interpreted as mediation or as the proportion of an association explained by renal dysfunction.

Sixth, the primary association and prediction analyses treated occurrence of each cardiovascular outcome during the available observation period as a binary endpoint. They did not estimate absolute risk at a prespecified 5-year or 10-year horizon and did not explicitly account for variation in follow-up duration. Participants who died without a recorded target event remained classified as noncases, and death was not modeled as a competing event. Consequently, the reported odds ratios, ROC-AUCs, Brier scores, and calibration measures should be interpreted as applying to the study-specific available observation period rather than as fixed-horizon clinical-risk estimates. Although the exploratory Cox analyses incorporated event timing and censored noncases at the earlier of death or the project-level proxy end date, the proxy end date was derived from the maximum observed hospital diagnosis-record date in the project extract rather than from a separately documented follow-up schedule, and the analyses did not constitute a formal competing-risk analysis. Seventh, the locked test sets were internal holdout samples rather than independent external-validation cohorts. The same overall source cohort and data-generating environment underlay training and testing, and performance may differ across populations, clinical settings, outcome definitions, and proteomic platforms. Classification thresholds were statistically frozen before test evaluation, but they were not derived from clinical decision consequences and do not establish clinical utility (Supplementary Table S12). Decision-curve analysis may be more informative in future studies after clinically meaningful risk horizons, intervention thresholds, and competing clinical strategies have been predefined. Finally, MG53 and MG29 could not be evaluated because corresponding measurements were not available in the analyzed UK Biobank proteomics release. The seven proteins examined here should therefore not be regarded as representing the complete excitation–contraction coupling system.

Future studies should first evaluate the reproducibility of the HRC and ITPR1 associations in independent prospective cohorts with comparable outcome definitions and should assess whether results are consistent across ancestry groups, proteomic platforms, and absolute or orthogonal measurement methods. Repeated protein measurements could help distinguish stable interindividual differences from changes associated with emerging subclinical disease. Tissue, cellular, genetic, and experimental studies are needed to determine whether circulating HRC or ITPR1 reflects altered myocardial calcium handling, tissue injury, clearance, or other systemic processes. Prediction research should evaluate broader combinations of EC7-related, established cardiovascular, inflammatory, renal, and myocardial-stress markers and should incorporate predefined clinical decision thresholds before claims of practical utility are made.

In conclusion, circulating excitation–contraction coupling proteins showed outcome-specific associations with incident cardiovascular disease, with the strongest and most consistent evidence involving HRC and, to a lesser extent, ITPR1 in heart failure. However, the seven-protein panel provided limited discrimination on its own and no multiplicity-adjusted incremental predictive improvement beyond clinical factors, while established cardiovascular comparator proteins performed better. These findings support further investigation of excitation–contraction coupling biology in heart-failure development but do not establish causal effects, external validity, or current clinical utility.

## Data Availability

The data analyzed in this study is subject to the following licenses/restrictions: UK Biobank application number 1034464. Requests to access these datasets should be directed to https://www.ukbiobank.ac.uk/.
