# Supplementary material for: Circulating excitation–contraction coupling proteins and incident cardiovascular outcomes: associations and incremental predictive performance in the UK Biobank
Source: Front Cardiovasc Med. 2026 Aug 12;13:1841253. doi: 10.3389/fcvm.2026.1841253 (PMC13507679; doi:10.3389/fcvm.2026.1841253)
Supplement: Supplementary file 1 [file Datasheet1.pdf]

Supplementary materials

Circulating Excitation–Contraction Coupling Proteins and Incident Cardiovascular Outcomes: Associations and Incremental Predictive Performance in the UK Biobank

Dan Hu[1, #], Yang Gao[1, #], Jing Wang[1], Quan Guo[1], Zhuocheng Shi[1], Donghui Chen[1], Dongsheng Xia[1], Muwei Li[1], Xiaohu Wang[1, *], Bingchuan Geng[1,2, *]

[1]*Fuwai Central China Cardiovascular Hospital, Central China Fuwai Hospital of Zhengzhou University, Zhengzhou 450000, China*

[2]*Henan Provincial Clinical Research Center for Cardiovascular Disease, Zhengzhou 450000, China*

[*]Corresponding authors.
E-mail addresses: Bingchuangeng@163.com (Bingchuan Geng); wxhwyl@126.com (Xiaohu Wang)
[#]These authors have contributed equally to this work.

The following supplementary materials are included in this file:

Figure S1-S7

Table S1-S12

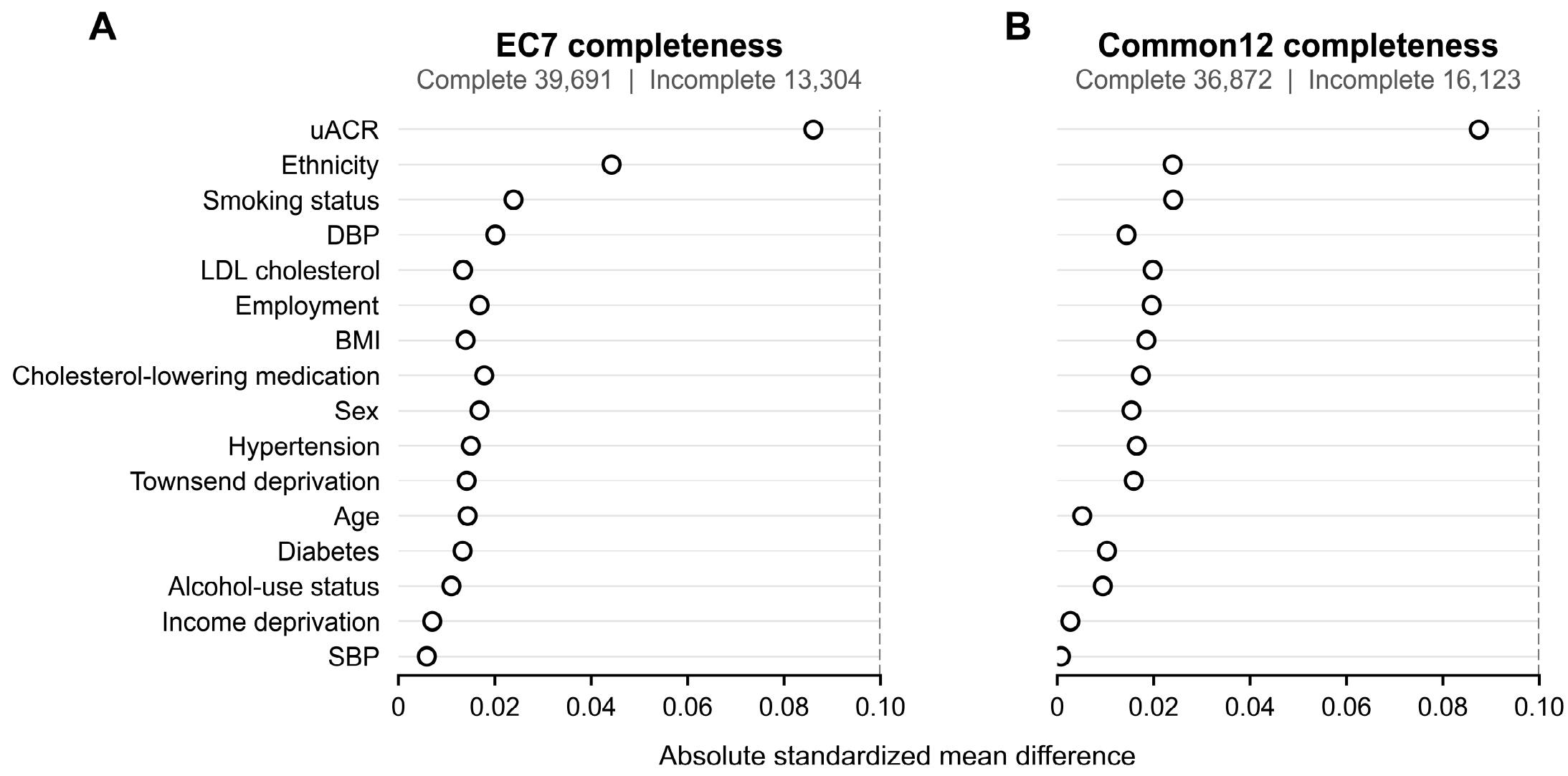

Figure S1. Baseline balance according to protein-panel completeness.

(A) Participants with complete versus incomplete EC7 measurements. (B) Participants with complete versus incomplete Common12 measurements. Points indicate absolute standardized mean differences in baseline characteristics; the dashed vertical line denotes 0.10.

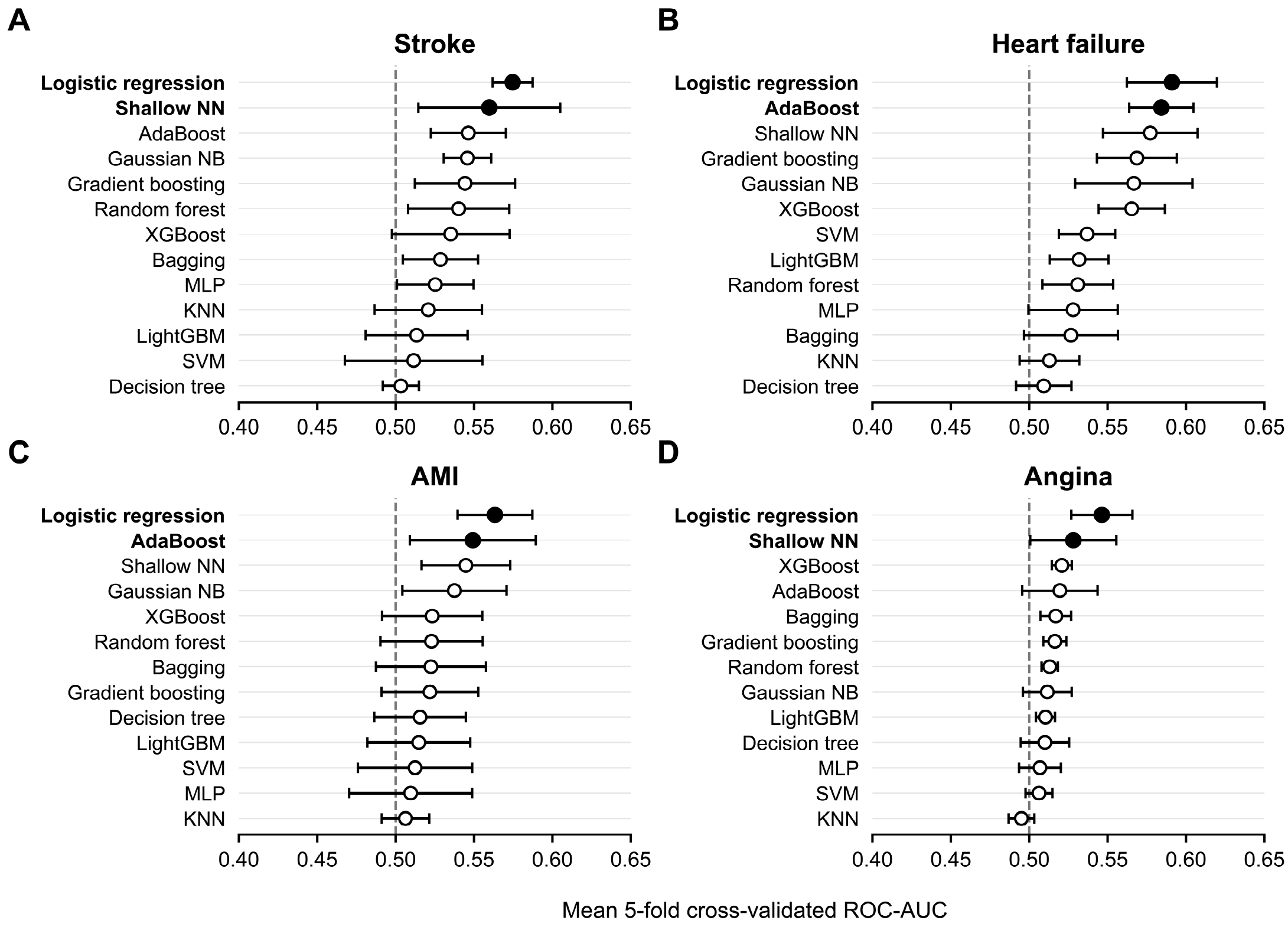

Figure S2. Training-set screening of candidate EC7 prediction algorithms.

(A) Stroke. (B) Heart failure. (C) Acute myocardial infarction. (D) Angina. Points indicate mean five-fold cross-validated ROC-AUC, and horizontal bars indicate ±1 standard deviation. Filled circles identify the two highest-ranked algorithms advanced to nested tuning; the dashed vertical line denotes ROC-AUC=0.50.

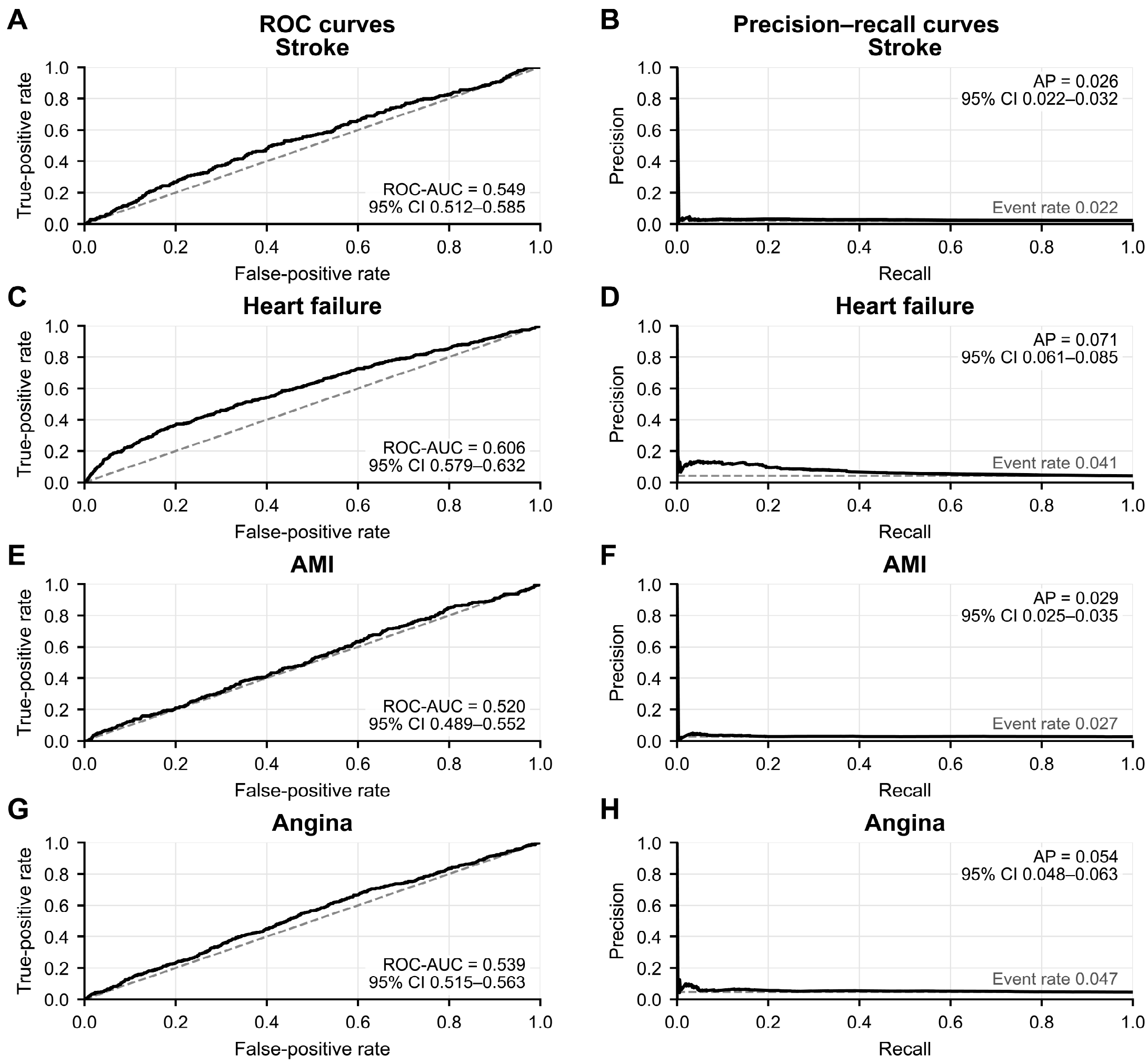

Figure S3. Locked-test discrimination of the final EC7 prediction models.

(A) Stroke receiver operating characteristic curve. (B) Stroke precision–recall curve. (C) Heart-failure receiver operating characteristic curve. (D) Heart-failure precision–recall curve. (E) Acute myocardial infarction receiver operating characteristic curve. (F) Acute myocardial infarction precision–recall curve. (G) Angina receiver operating characteristic curve. (H) Angina precision–recall curve. ROC panels report ROC-AUC, and precision–recall panels report average precision with bootstrap 95% confidence intervals. Dashed lines indicate random discrimination or the corresponding locked-test event rate.

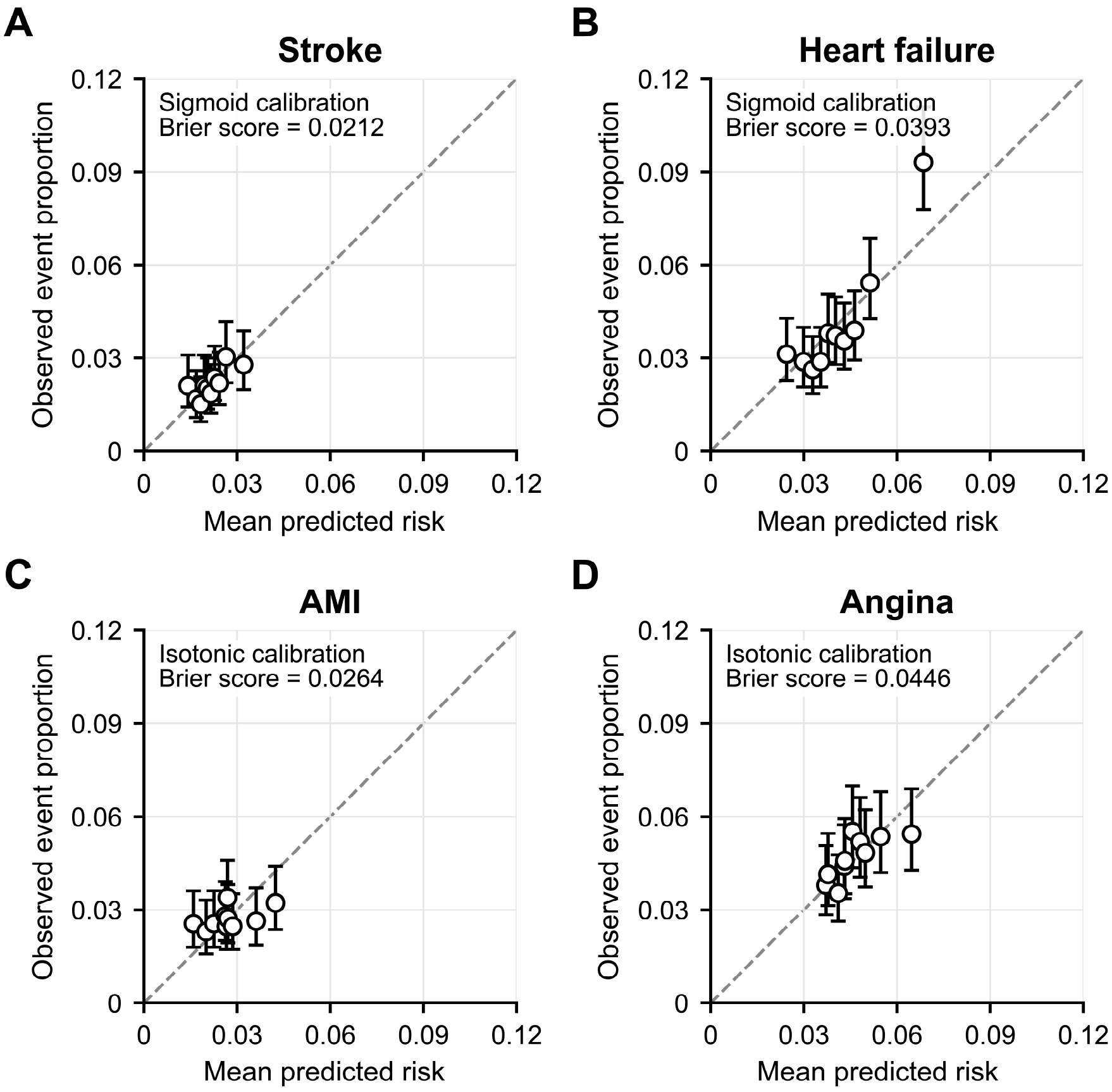

Figure S4. Locked-test calibration of the final EC7 prediction models.

(A) Stroke. (B) Heart failure. (C) Acute myocardial infarction. (D) Angina. Points indicate observed event proportions versus mean calibrated predicted risks across 10 approximately equal-sized groups, with vertical bars showing 95% confidence intervals. The dashed diagonal line denotes ideal calibration, and the calibrated Brier score is shown in each panel.

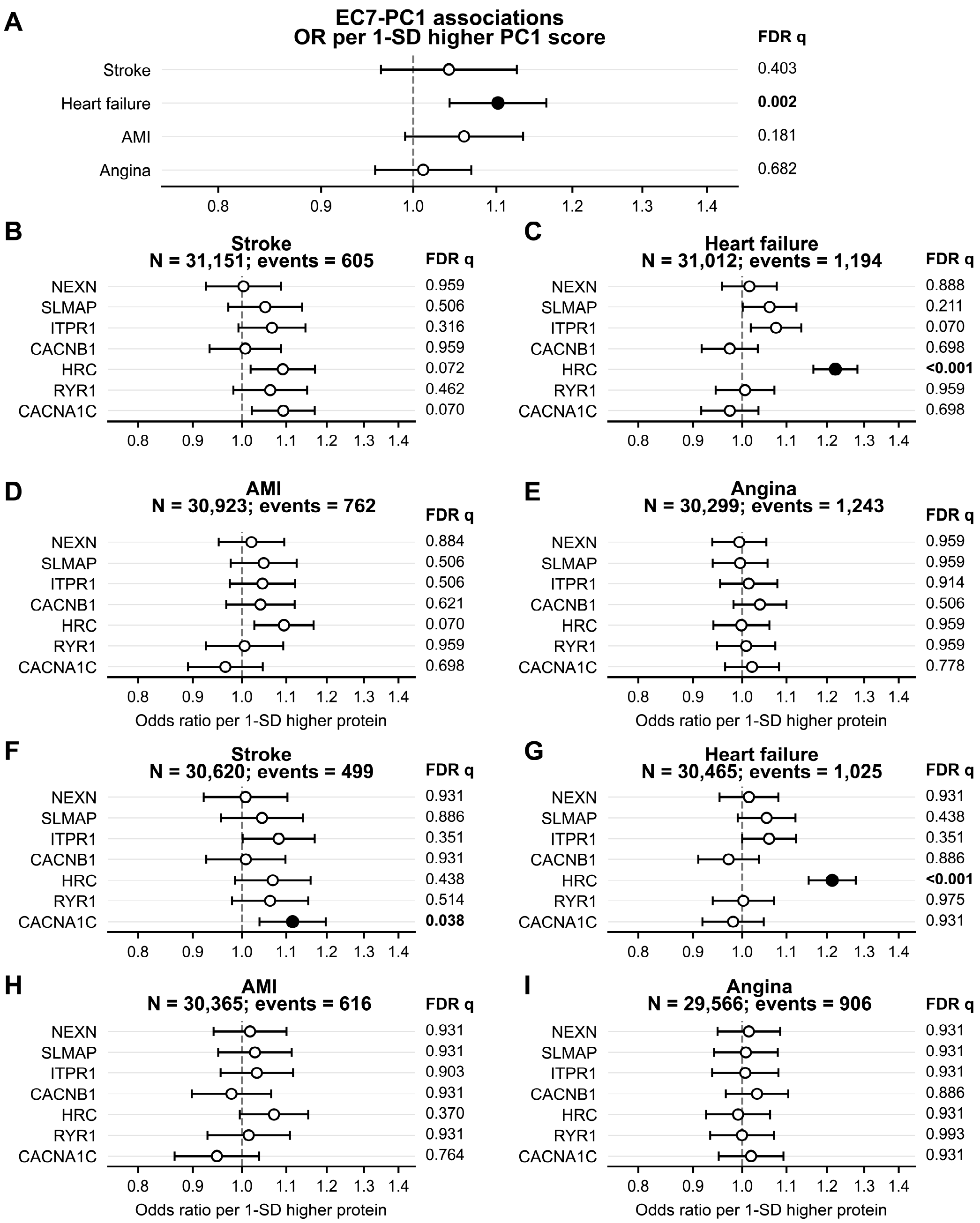

Figure S5. Supportive EC7 principal-component and landmark association analyses.

(A) Clinically adjusted associations of the standardized EC7 first principal component with the four cardiovascular outcomes. (B) Two-year landmark analysis for stroke. (C) Two-year landmark analysis for heart failure. (D) Two-year landmark analysis for acute myocardial infarction. (E) Two-year landmark analysis for angina. (F) Five-year landmark analysis for stroke. (G) Five-year landmark analysis for heart failure. (H) Five-year landmark analysis for acute myocardial infarction. (I) Five-year landmark analysis for angina. Points indicate odds ratios per 1-standard-deviation higher score or relative protein level, with horizontal lines showing 95% confidence intervals. Filled circles indicate Benjamini – Hochberg false-discovery-rate q<0.05 within the corresponding testing family.

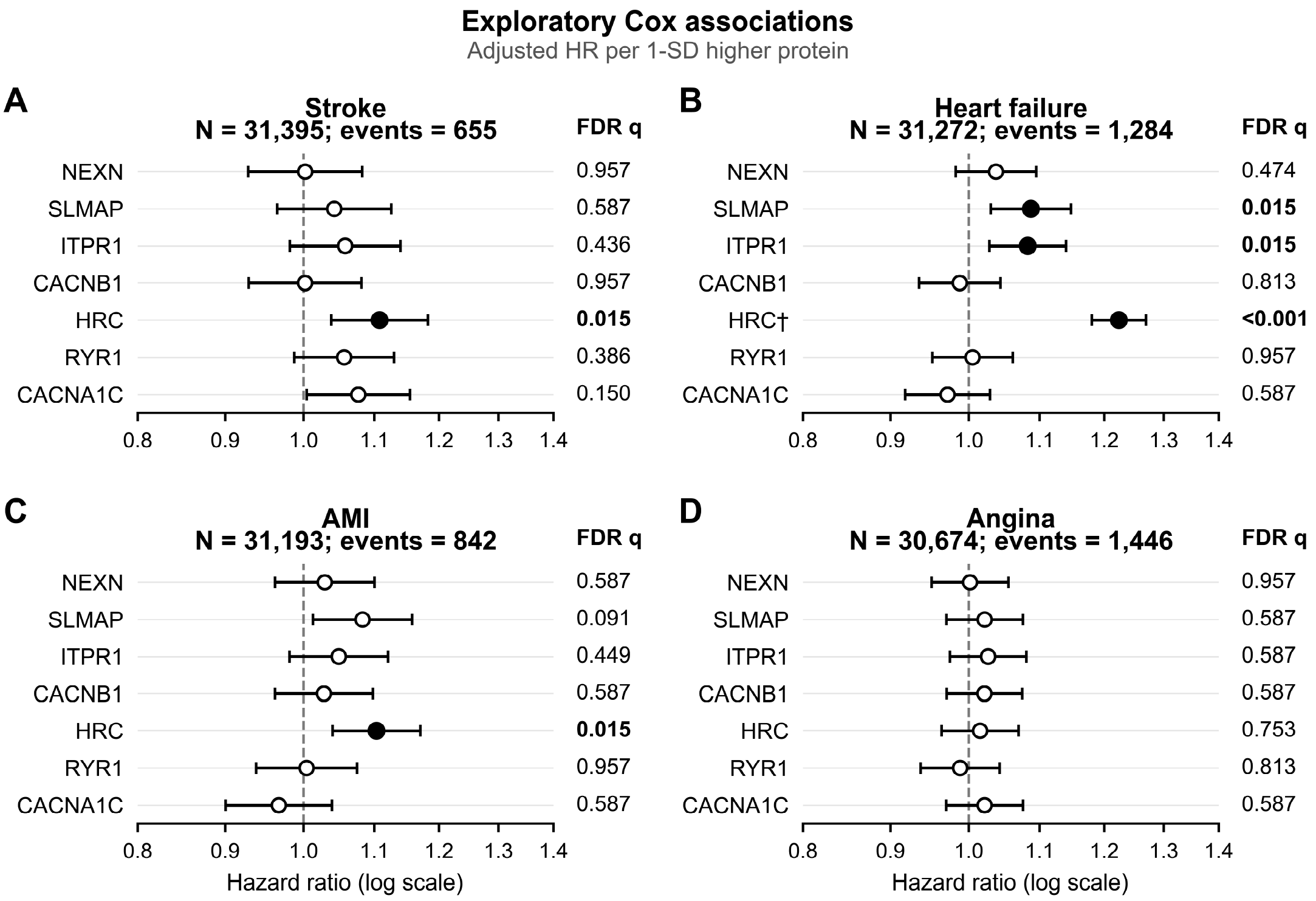

Figure S6. Exploratory Cox associations of EC7 proteins with incident cardiovascular outcomes.

(A) Stroke. (B) Heart failure. (C) Acute myocardial infarction. (D) Angina. Hazard ratios are reported per 1-standard-deviation higher relative protein level from clinically adjusted Cox proportional-hazards models. Horizontal lines indicate 95% confidence intervals, and the dashed vertical line denotes a hazard ratio of 1. Filled circles indicate Benjamini–Hochberg false-discovery-rate q<0.05; the dagger identifies a proportional-hazards diagnostic that remained significant after correction.

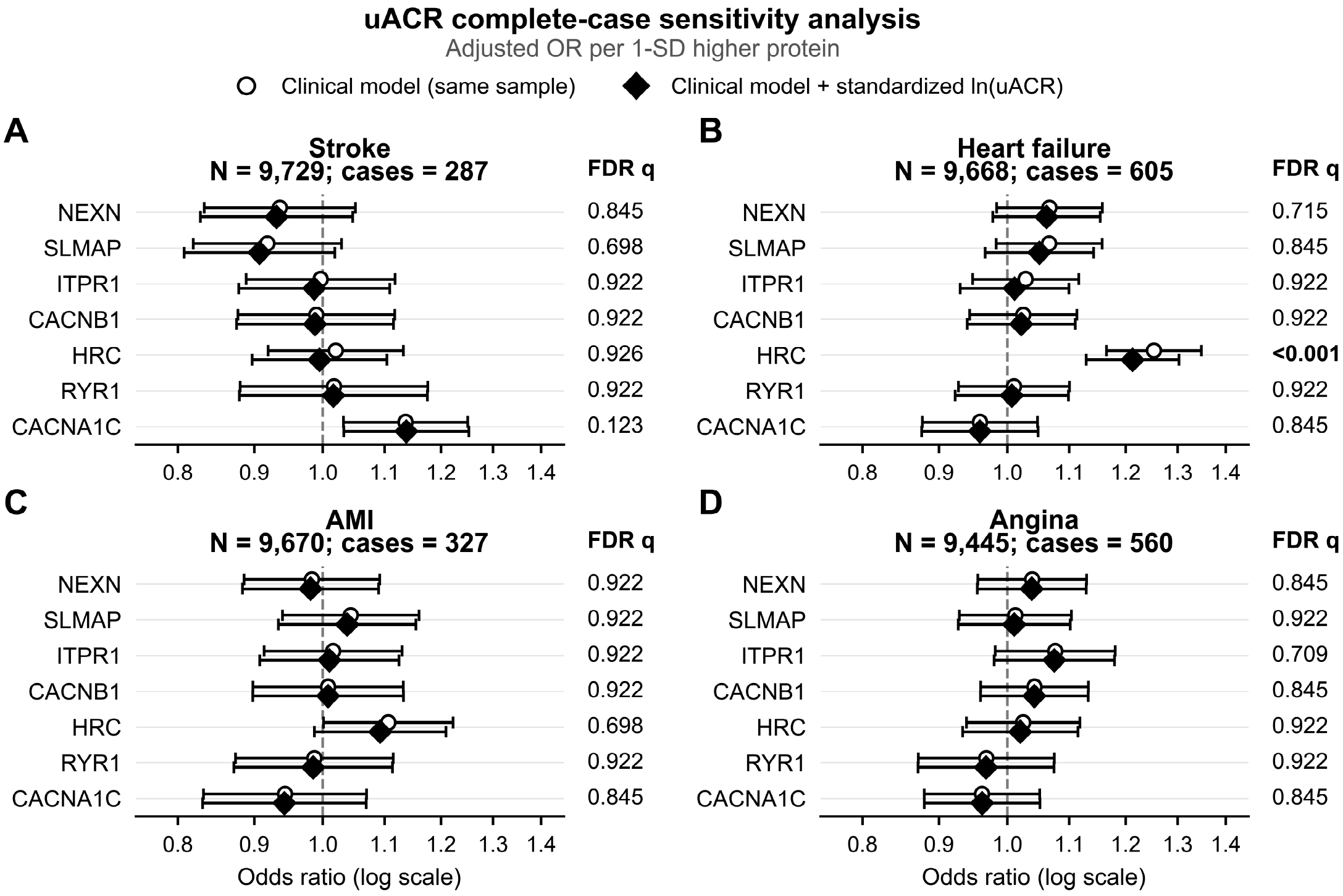

Figure S7. Complete-case uACR sensitivity analysis of EC7 protein associations.

(A) Stroke. (B) Heart failure. (C) Acute myocardial infarction. (D) Angina. Open circles indicate clinically adjusted estimates re-estimated in the uACR-complete sample, and filled diamonds indicate estimates additionally adjusted for standardized log-transformed uACR. Odds ratios are reported per 1-standard-deviation higher relative protein level, with horizontal lines showing 95% confidence intervals. Bold q-values indicate Benjamini–Hochberg false-discovery-rate q<0.05 for the uACR-adjusted associations.

Table S1. Definitions and derivation of cardiovascular outcomes, EC7 proteins, and clinical covariates

Variable	Operational definition	UK Biobank field(s) or source	Coding or unit	Analysis role
**Incident cardiovascular outcomes**				
**Ischemic or unspecified stroke**	Qualifying ICD-10 diagnosis with prefix I63 or I64; hemorrhagic stroke codes were not included.	UK Biobank Fields 41270 and 41280.	Binary: incident event = 1; eligible noncase = 0.	Primary outcome.
**Heart failure**	Qualifying ICD-10 diagnosis with prefix I50.	UK Biobank Fields 41270 and 41280.	Binary: incident event = 1; eligible noncase = 0.	Primary outcome.
**Acute myocardial infarction**	Qualifying ICD-10 diagnosis with prefix I21.	UK Biobank Fields 41270 and 41280.	Binary: incident event = 1; eligible noncase = 0.	Primary outcome.
**Angina**	Qualifying ICD-10 diagnosis with prefix I20.	UK Biobank Fields 41270 and 41280.	Binary: incident event = 1; eligible noncase = 0.	Primary outcome.
**EC7 proteins**				
**NEXN**	Relative protein level corresponding to the NEXN gene symbol.	Canonical proteomics dataset.	For association analyses, estimates were expressed per 1-SD higher value using the full outcome-specific EC7 cohort SD.	Primary exposure; component of the EC7 prediction feature set.

Variable	Operational definition	UK Biobank field(s) or source	Coding or unit	Analysis role
**SLMAP**	Relative protein level corresponding to the SLMAP gene symbol.	Canonical proteomics dataset; variable slmap.	For association analyses, estimates were expressed per 1-SD higher value using the full outcome-specific EC7 cohort SD.	Primary exposure; component of the EC7 prediction feature set.
**ITPR1**	Relative protein level corresponding to the ITPR1 gene symbol.	Canonical proteomics dataset; variable itpr1.	For association analyses, estimates were expressed per 1-SD higher value using the full outcome-specific EC7 cohort SD.	Primary exposure; component of the EC7 prediction feature set.
**CACNB1**	Relative protein level corresponding to the CACNB1 gene symbol.	Canonical proteomics dataset; variable cacnb1.	For association analyses, estimates were expressed per 1-SD higher value using the full outcome-specific EC7 cohort SD.	Primary exposure; component of the EC7 prediction feature set.
**HRC**	Relative protein level corresponding to the HRC gene symbol.	Canonical proteomics dataset; variable hrc.	For association analyses, estimates were expressed per 1-SD higher value using the full outcome-specific EC7 cohort SD.	Primary exposure; component of the EC7 prediction feature set.
**RYR1**	Relative protein level corresponding to the RYR1 gene symbol.	Canonical proteomics dataset; variable ryr1.	For association analyses, estimates were expressed per 1-SD higher value using the full outcome-specific EC7 cohort SD.	Primary exposure; component of the EC7 prediction feature set.
**CACNA1C**	Relative protein level corresponding to the CACNA1C gene symbol.	Canonical proteomics dataset; variable cacna1c.	For association analyses, estimates were expressed per 1-SD higher value using the full outcome-specific EC7 cohort SD.	Primary exposure; component of the EC7 prediction feature set.
**Primary clinical covariates**				

Variable	Operational definition	UK Biobank field(s) or source	Coding or unit	Analysis role
**Age**	Age at baseline assessment.	UK Biobank Field 21022.	Continuous, years.	Primary adjustment covariate.
**Sex**	Sex recorded at baseline.	Baseline dataset; UK Biobank Field 31 provenance.	Female or male; female was the model reference.	Primary adjustment covariate.
**Body mass index**	Body mass index measured at baseline.	Baseline dataset; UK Biobank Field 21001 provenance.	Continuous, kg/m^2.	Primary adjustment covariate.
**Smoking status**	Baseline smoking status.	UK Biobank Field 20116, baseline instance.	0 = never, 1 = previous, 2 = current; never was the model reference.	Primary adjustment covariate.
**Systolic blood pressure**	Mean of available automated readings; mean manual readings were used when automated measurements were unavailable.	Automated: Field 4080; manual: Field 93.	Continuous, mmHg.	Primary adjustment covariate.
**LDL cholesterol**	Baseline low-density lipoprotein cholesterol.	Baseline dataset; UK Biobank Field 30780 provenance.	Continuous, mmol/L.	Primary adjustment covariate.
**Hypertension**	Positive when doctor-reported hypertension, blood-pressure medication use, or self-reported hypertension was present.	Field 6150 code 4; Fields 6153/6177 code 2; Field 20002 codes 1065 or 1072.	Binary: 1 = hypertension; 0 = valid negative response; otherwise missing.	Primary adjustment covariate.

Variable	Operational definition	UK Biobank field(s) or source	Coding or unit	Analysis role
**Diabetes mellitus**	Positive when non-gestational doctor-diagnosed diabetes, insulin use, or self-reported diabetes was present; gestational-only diabetes was excluded.	Field 2443 code 1; Field 4041 code 1 where available; Fields 6153/6177 code 3; Field 20002 codes 1220, 1222, or	Binary: 1 = diabetes; 0 = valid negative response; otherwise missing.	Primary adjustment covariate.
**Blood-pressure medication**	Self-reported use of medication for blood pressure.	UK Biobank Fields 6153 and 6177, code 2.	Binary: 1 = reported use; 0 = otherwise.	Primary adjustment covariate.
**Cholesterol-lowering medication**	Self-reported use of medication for lowering cholesterol; not restricted to statins.	UK Biobank Fields 6153 and 6177, code 1.	Binary: 1 = reported use; 0 = valid negative response; otherwise missing.	Primary adjustment covariate.
**Income deprivation score**	Income deprivation score for England, used without further recoding.	UK Biobank Field 26411.	Continuous score.	Primary adjustment covariate.
**Sensitivity variable**				
**Urinary albumin-to-creatinine ratio**	Urinary albumin divided by urinary creatinine and multiplied by 1,000.	Urinary albumin: Field 30500; urinary creatinine: Field 30510.	mg/mmol; natural-log transformed and standardized per 1-SD higher value within each outcome-specific complete-case sample.	Additional covariate in the uACR sensitivity analysis only.

Note: Incident outcomes were defined from hospital inpatient ICD-10 diagnosis codes and corresponding diagnosis dates. Diagnoses recorded on or before baseline were classified as prevalent, whereas the earliest qualifying post-baseline diagnosis recorded on or before 31 October 2022 defined an incident event. The latter date was used as the project-level proxy end date and was derived from the maximum observed hospital diagnosis-record date available in the project extract. Participants who died without a recorded qualifying event before death remained classified as noncases in the primary binary analyses. For time-to-event analyses, follow-up ended at the first qualifying event date for cases and at the earlier of death or the proxy end date for noncases. Protein measurements were analyzed as relative Olink values and were not interpreted as absolute plasma concentrations.

Table S2. Protein panel composition and measurement completeness in the source proteomics cohort

Panel A. Individual protein measurement completeness

Protein panel	Protein	Available measurements, n (%)	Missing measurements, n (%)
EC7	**NEXN**	44,802 (84.54)	8,193 (15.46)
	**SLMAP**	44,573 (84.11)	8,422 (15.89)
	**ITPR1**	43,574 (82.22)	9,421 (17.78)
	**CACNB1**	43,322 (81.75)	9,673 (18.25)
	**HRC**	43,574 (82.22)	9,421 (17.78)
	**RYR1**	43,296 (81.70)	9,699 (18.30)
	**CACNA1C**	43,322 (81.75)	9,673 (18.25)
Established comparator proteins	**NT-proBNP**	51,494 (97.17)	1,501 (2.83)
	**GDF15**	52,130 (98.37)	865 (1.63)
	**MMP12**	51,535 (97.25)	1,460 (2.75)
	**HAVCR1**	51,859 (97.86)	1,136 (2.14)
	**TNFRSF10B**	51,096 (96.42)	1,899 (3.58)

Panel B. Measurement-complete protein sets

Measurement-complete set	Definition	Required proteins, n	Complete participants, n (%)	Incomplete participants, n (%)
**Complete EC7 panel**	All seven EC7 protein measurements were non-missing.	7	39,691 (74.90)	13,304 (25.10)
**Complete established**	All five established comparator protein measurements were non-	5	49,187 (92.81)	3,808 (7.19)
**Complete 12-protein set**	All seven EC7 and all five established comparator protein	12	36,872 (69.58)	16,123 (30.42)

Note: Percentages were calculated using the analysis source proteomics cohort of 52,995 participants as the denominator. Protein-panel completeness was assessed before outcome-specific exclusions, clinical-covariate complete-case restriction, and train–test partitioning. A complete panel required nonmissing measurements for every constituent protein; no imputation was used to define panel completeness.

Table S3. Locked test-set performance and calibration of the EC7-only prediction models

Panel A. Discrimination and overall probabilistic performance

Outcome	Test n	Cases, n (%)	Calibration	Raw ROC-AUC	Calibrated ROC-AUC	Raw AP	Calibrated AP
**Stroke**	11,856	257 (2.17)	Sigmoid	0.549 (0.512–0.585)	0.549 (0.512–0.585)	0.026 (0.022–0.032)	0.026 (0.022–0.032)
**Heart failure**	11,813	487 (4.12)	Sigmoid	0.606 (0.579–0.632)	0.606 (0.579–0.632)	0.071 (0.061–0.085)	0.071 (0.061–0.085)
**AMI**	11,782	319 (2.71)	Isotonic	0.520 (0.489–0.552)	0.518 (0.487–0.549)	0.029 (0.025–0.035)	0.029 (0.025–0.033)
**Angina**	11,591	542 (4.68)	Isotonic	0.539 (0.515–0.563)	0.536 (0.511–0.559)	0.054 (0.048–0.063)	0.052 (0.046–0.059)

Panel B. Calibration and overall prediction error

Outcome	Brier score	Null Brier score	Brier skill score	Log loss	Calibration intercept	Calibration slope
**Stroke**	0.0212 (0.0186–0.0238)	0.0212 (0.0186–0.0239)	0.0005 (-0.0010–0.0017)	0.1042 (0.0938–0.1148)	-1.028	0.729
**Heart failure**	0.0393 (0.0359–0.0425)	0.0395 (0.0362–0.0428)	0.0064 (0.0031–0.0097)	0.1690 (0.1575–0.1802)	0.564	1.180
**AMI**	0.0264 (0.0237–0.0293)	0.0263 (0.0237–0.0292)	-0.0014 (-0.0031–0.0002)	0.1257 (0.1154–0.1368)	-3.382	0.055
**Angina**	0.0446 (0.0410–0.0481)	0.0446 (0.0410–0.0481)	-0.0001 (-0.0020–0.0016)	0.1896 (0.1777–0.2018)	-2.615	0.131

Panel C. Performance at the frozen probability threshold

Outcome	Frozen threshold	Sensitivity	Specificity	PPV	NPV	Balanced accuracy
**Stroke**	0.0220	0.498 (0.436–0.561)	0.590 (0.581–0.599)	0.026 (0.022–0.031)	0.982 (0.978–0.985)	0.544 (0.513–0.576)
**Heart failure**	0.0431	0.499 (0.458–0.542)	0.661 (0.652–0.669)	0.060 (0.053–0.067)	0.968 (0.965–0.972)	0.580 (0.558–0.602)
**AMI**	0.0234	0.762 (0.712–0.806)	0.276 (0.268–0.285)	0.028 (0.025–0.032)	0.977 (0.971–0.982)	0.519 (0.494–0.542)
**Angina**	0.0446	0.507 (0.462–0.549)	0.553 (0.544–0.562)	0.053 (0.047–0.059)	0.958 (0.953–0.963)	0.530 (0.508–0.551)

Note: Results are from the one-time evaluation of the outcome-specific locked test sets using the selected EC7-only logistic-regression models. Sigmoid calibration was used for stroke and heart failure, and isotonic calibration for acute myocardial infarction and angina; calibration methods and classification thresholds were fixed using training data. Confidence intervals are percentile 95% intervals from 2,000 bootstrap replicates, whereas calibration intercept and slope are reported as point estimates. Lower Brier scores and log loss and higher Brier skill scores indicate better performance; threshold-based metrics correspond to the frozen outcome-specific thresholds.

Table S4. Locked test-set performance and secondary comparisons across EC7, Comparator5, and clinical feature sets

Panel A. Absolute locked test-set performance of the five prespecified feature sets

Outcome	Feature set	Test n	Cases, n (%)	ROC-AUC (95% CI)	AP (95% CI)	Brier score (95% CI)	Brier skill score (95% CI)	Log loss (95% CI)	Calibration intercept	Calibration
Stroke	EC7 proteins only	10,986	241 (2.19%)	0.546 (0.510 to 0.583)	0.026 (0.023 to 0.031)	0.02146 (0.02145 to 0.02146)	-0.0001 (-0.0003 to 0.0001)	0.10553 (0.10542 to 0.10563)	11.679	3.950
Stroke	Established comparator proteins only	10,986	241 (2.19%)	0.733 (0.702 to 0.763)	0.059 (0.049 to 0.075)	0.02122 (0.02105 to 0.02137)	0.0110 (0.0038 to 0.0190)	0.09948 (0.09748 to 0.10150)	0.303	1.003
Stroke	Clinical covariates only	10,986	241 (2.19%)	0.750 (0.721 to 0.779)	0.063 (0.052 to 0.083)	0.02104 (0.02091 to 0.02117)	0.0193 (0.0132 to 0.0253)	0.09701 (0.09518 to 0.09886)	0.714	1.196
Stroke	Clinical covariates + EC7 proteins	10,986	241 (2.19%)	0.745 (0.715 to 0.774)	0.059 (0.050 to 0.076)	0.02108 (0.02095 to 0.02120)	0.0176 (0.0117 to 0.0234)	0.09743 (0.09556 to 0.09927)	0.578	1.157
Stroke	Clinical covariates + established comparator	10,986	241 (2.19%)	0.765 (0.738 to 0.794)	0.068 (0.057 to 0.087)	0.02098 (0.02081 to 0.02114)	0.0220 (0.0146 to 0.0302)	0.09605 (0.09401 to 0.09792)	0.591	1.169
Heart failure	EC7 proteins only	10,990	457 (4.16%)	0.608 (0.581 to 0.636)	0.071 (0.062 to 0.083)	0.03976 (0.03972 to 0.03979)	0.0025 (0.0016 to 0.0034)	0.17186 (0.17148 to 0.17223)	7.758	3.592
Heart failure	Established comparator proteins only	10,990	457 (4.16%)	0.808 (0.787 to 0.827)	0.216 (0.187 to 0.252)	0.03631 (0.03535 to 0.03721)	0.0889 (0.0664 to 0.1129)	0.14448 (0.13991 to 0.14881)	0.083	0.976
Heart failure	Clinical covariates only	10,990	457 (4.16%)	0.788 (0.768 to 0.807)	0.155 (0.135 to 0.185)	0.03745 (0.03690 to 0.03795)	0.0604 (0.0479 to 0.0742)	0.15002 (0.14650 to 0.15357)	0.196	1.058
Heart failure	Clinical covariates + EC7 proteins	10,990	457 (4.16%)	0.794 (0.774 to 0.813)	0.164 (0.142 to 0.195)	0.03728 (0.03668 to 0.03781)	0.0647 (0.0513 to 0.0796)	0.14889 (0.14537 to 0.15235)	0.215	1.075
Heart failure	Clinical covariates + established comparator	10,990	457 (4.16%)	0.831 (0.811 to 0.849)	0.249 (0.216 to 0.289)	0.03525 (0.03427 to 0.03619)	0.1156 (0.0919 to 0.1400)	0.13870 (0.13425 to 0.14297)	0.157	1.056
Acute myocardial	EC7 proteins only	10,935	295 (2.70%)	0.536 (0.501 to 0.570)	0.032 (0.028 to 0.040)	0.02625 (0.02624 to 0.02625)	0.0001 (0.0000 to 0.0002)	0.12402 (0.12397 to 0.12408)	20.000	6.625

Outcome	Feature set	Test n	Cases, n (%)	ROC-AUC (95% CI)	AP (95% CI)	Brier score (95% CI)	Brier skill score (95% CI)	Log loss (95% CI)	Calibration intercept	Calibration
Acute myocardial	Established comparator proteins only	10,935	295 (2.70%)	0.695 (0.662 to 0.723)	0.059 (0.051 to 0.073)	0.02604 (0.02584 to 0.02623)	0.0081 (0.0009 to 0.0158)	0.11884 (0.11680 to 0.12105)	-0.183	0.886
Acute myocardial	Clinical covariates only	10,935	295 (2.70%)	0.702 (0.674 to 0.728)	0.058 (0.050 to 0.072)	0.02593 (0.02575 to 0.02611)	0.0121 (0.0055 to 0.0192)	0.11730 (0.11516 to 0.11942)	-0.383	0.889
Acute myocardial	Clinical covariates + EC7 proteins	10,935	295 (2.70%)	0.699 (0.671 to 0.727)	0.055 (0.048 to 0.068)	0.02597 (0.02579 to 0.02614)	0.0106 (0.0042 to 0.0174)	0.11765 (0.11555 to 0.11972)	-0.444	0.871
Acute myocardial	Clinical covariates + established comparator	10,935	295 (2.70%)	0.729 (0.701 to 0.754)	0.065 (0.056 to 0.079)	0.02587 (0.02564 to 0.02609)	0.0146 (0.0062 to 0.0233)	0.11560 (0.11341 to 0.11795)	-0.257	0.929
Angina	EC7 proteins only	10,802	509 (4.71%)	0.537 (0.511 to 0.562)	0.054 (0.050 to 0.062)	0.04489 (0.04488 to 0.04490)	0.0002 (0.0000 to 0.0004)	0.18985 (0.18977 to 0.18994)	15.624	6.249
Angina	Established comparator proteins only	10,802	509 (4.71%)	0.677 (0.653 to 0.699)	0.091 (0.081 to 0.104)	0.04432 (0.04395 to 0.04469)	0.0129 (0.0046 to 0.0211)	0.18238 (0.17993 to 0.18492)	-0.216	0.882
Angina	Clinical covariates only	10,802	509 (4.71%)	0.728 (0.706 to 0.749)	0.111 (0.099 to 0.130)	0.04357 (0.04312 to 0.04401)	0.0296 (0.0197 to 0.0396)	0.17549 (0.17245 to 0.17861)	-0.120	0.971
Angina	Clinical covariates + EC7 proteins	10,802	509 (4.71%)	0.727 (0.705 to 0.748)	0.112 (0.099 to 0.129)	0.04360 (0.04313 to 0.04407)	0.0289 (0.0185 to 0.0395)	0.17563 (0.17244 to 0.17889)	-0.234	0.924
Angina	Clinical covariates + established comparator	10,802	509 (4.71%)	0.734 (0.712 to 0.755)	0.117 (0.103 to 0.135)	0.04346 (0.04296 to 0.04400)	0.0320 (0.0200 to 0.0432)	0.17457 (0.17131 to 0.17806)	-0.184	0.946

Panel B. Prespecified incremental, biomarker-only and clinical-biomarker paired comparisons

Outcome	Comparison family	Comparison (model A vs model B)	Metric	Difference (95% CI)	Bootstrap P value	FDR-adjusted q
Stroke	Primary incremental	Clinical + EC7 vs clinical only	ROC-AUC	-0.0059 (-0.0131 to +0.0017)	0.145	0.193
Stroke	Primary incremental	Clinical + EC7 vs clinical only	AP	-0.0039 (-0.0117 to +0.0009)	0.113	0.217
Stroke	Primary incremental	Clinical + EC7 vs clinical only	Brier score	+0.00004 (0.00000 to +0.00007)	0.060	0.183
Stroke	Primary incremental	Clinical + EC7 vs clinical only	Brier skill score	-0.0017 (-0.0035 to +0.0001)	0.060	0.183
Stroke	Primary incremental	Clinical + EC7 vs clinical only	Log loss	+0.00042 (-0.00007 to +0.00086)	0.089	0.178
Stroke	Biomarker only	EC7 only vs established comparator proteins only	ROC-AUC	-0.1867 (-0.2252 to -0.1473)	<0.001	<0.001

Outcome	Comparison family	Comparison (model A vs model B)	Metric	Difference (95% CI)	Bootstrap P value	FDR-adjusted q
Stroke	Biomarker only	EC7 only vs established comparator proteins only	AP	-0.0333 (-0.0477 to -0.0237)	<0.001	<0.001
Stroke	Biomarker only	EC7 only vs established comparator proteins only	Brier score	+0.00024 (+0.00009 to +0.00041)	0.004	0.005
Stroke	Biomarker only	EC7 only vs established comparator proteins only	Brier skill score	-0.0111 (-0.0189 to -0.0040)	0.004	0.005
Stroke	Biomarker only	EC7 only vs established comparator proteins only	Log loss	+0.00605 (+0.00408 to +0.00802)	<0.001	<0.001
Stroke	Clinical biomarker	Clinical + EC7 vs clinical + established comparator	ROC-AUC	-0.0204 (-0.0333 to -0.0085)	0.003	0.004
Stroke	Clinical biomarker	Clinical + EC7 vs clinical + established comparator	AP	-0.0086 (-0.0175 to -0.0001)	0.048	0.064
Stroke	Clinical biomarker	Clinical + EC7 vs clinical + established comparator	Brier score	+0.00009 (+0.00001 to +0.00019)	0.040	0.080
Stroke	Clinical biomarker	Clinical + EC7 vs clinical + established comparator	Brier skill score	-0.0044 (-0.0089 to -0.0003)	0.040	0.080
Stroke	Clinical biomarker	Clinical + EC7 vs clinical + established comparator	Log loss	+0.00138 (+0.00052 to +0.00232)	0.003	0.004
Heart failure	Primary incremental	Clinical + EC7 vs clinical only	ROC-AUC	+0.0054 (+0.0006 to +0.0104)	0.028	0.112
Heart failure	Primary incremental	Clinical + EC7 vs clinical only	AP	+0.0084 (-0.0036 to +0.0202)	0.163	0.217
Heart failure	Primary incremental	Clinical + EC7 vs clinical only	Brier score	-0.00017 (-0.00038 to +0.00005)	0.137	0.183
Heart failure	Primary incremental	Clinical + EC7 vs clinical only	Brier skill score	+0.0042 (-0.0012 to +0.0096)	0.137	0.183
Heart failure	Primary incremental	Clinical + EC7 vs clinical only	Log loss	-0.00113 (-0.00214 to -0.00015)	0.023	0.092
Heart failure	Biomarker only	EC7 only vs established comparator proteins only	ROC-AUC	-0.2000 (-0.2303 to -0.1709)	<0.001	<0.001
Heart failure	Biomarker only	EC7 only vs established comparator proteins only	AP	-0.1450 (-0.1769 to -0.1182)	<0.001	<0.001
Heart failure	Biomarker only	EC7 only vs established comparator proteins only	Brier score	+0.00344 (+0.00257 to +0.00439)	<0.001	0.004
Heart failure	Biomarker only	EC7 only vs established comparator proteins only	Brier skill score	-0.0864 (-0.1101 to -0.0645)	<0.001	0.004
Heart failure	Biomarker only	EC7 only vs established comparator proteins only	Log loss	+0.02738 (+0.02329 to +0.03190)	<0.001	<0.001
Heart failure	Clinical biomarker	Clinical + EC7 vs clinical + established comparator	ROC-AUC	-0.0370 (-0.0498 to -0.0249)	<0.001	0.002
Heart failure	Clinical biomarker	Clinical + EC7 vs clinical + established comparator	AP	-0.0851 (-0.1127 to -0.0592)	<0.001	0.004
Heart failure	Clinical biomarker	Clinical + EC7 vs clinical + established comparator	Brier score	+0.00203 (+0.00137 to +0.00271)	<0.001	0.004
Heart failure	Clinical biomarker	Clinical + EC7 vs clinical + established comparator	Brier skill score	-0.0510 (-0.0679 to -0.0345)	<0.001	0.004
Heart failure	Clinical biomarker	Clinical + EC7 vs clinical + established comparator	Log loss	+0.01020 (+0.00752 to +0.01281)	<0.001	0.002
Acute myocardial	Primary incremental	Clinical + EC7 vs clinical only	ROC-AUC	-0.0023 (-0.0093 to +0.0049)	0.529	0.529
Acute myocardial	Primary incremental	Clinical + EC7 vs clinical only	AP	-0.0025 (-0.0064 to +0.0006)	0.114	0.217
Acute myocardial	Primary incremental	Clinical + EC7 vs clinical only	Brier score	+0.00004 (-0.00001 to +0.00008)	0.106	0.183

Outcome	Comparison family	Comparison (model A vs model B)	Metric	Difference (95% CI)	Bootstrap P value	FDR-adjusted q
Acute myocardial	Primary incremental	Clinical + EC7 vs clinical only	Brier skill score	-0.0015 (-0.0031 to +0.0003)	0.106	0.183
Acute myocardial	Primary incremental	Clinical + EC7 vs clinical only	Log loss	+0.00035 (-0.00017 to +0.00085)	0.199	0.265
Acute myocardial	Biomarker only	EC7 only vs established comparator proteins only	ROC-AUC	-0.1592 (-0.2028 to -0.1140)	<0.001	<0.001
Acute myocardial	Biomarker only	EC7 only vs established comparator proteins only	AP	-0.0275 (-0.0396 to -0.0166)	<0.001	<0.001
Acute myocardial	Biomarker only	EC7 only vs established comparator proteins only	Brier score	+0.00021 (+0.00002 to +0.00041)	0.032	0.032
Acute myocardial	Biomarker only	EC7 only vs established comparator proteins only	Brier skill score	-0.0080 (-0.0156 to -0.0008)	0.032	0.032
Acute myocardial	Biomarker only	EC7 only vs established comparator proteins only	Log loss	+0.00519 (+0.00298 to +0.00720)	<0.001	<0.001
Acute myocardial	Clinical biomarker	Clinical + EC7 vs clinical + established comparator	ROC-AUC	-0.0292 (-0.0419 to -0.0161)	<0.001	0.002
Acute myocardial	Clinical biomarker	Clinical + EC7 vs clinical + established comparator	AP	-0.0099 (-0.0180 to -0.0027)	0.013	0.026
Acute myocardial	Clinical biomarker	Clinical + EC7 vs clinical + established comparator	Brier score	+0.00011 (-0.00004 to +0.00024)	0.146	0.195
Acute myocardial	Clinical biomarker	Clinical + EC7 vs clinical + established comparator	Brier skill score	-0.0040 (-0.0091 to +0.0013)	0.146	0.195
Acute myocardial	Clinical biomarker	Clinical + EC7 vs clinical + established comparator	Log loss	+0.00206 (+0.00099 to +0.00312)	<0.001	0.002
Angina	Primary incremental	Clinical + EC7 vs clinical only	ROC-AUC	-0.0013 (-0.0030 to +0.0003)	0.134	0.193
Angina	Primary incremental	Clinical + EC7 vs clinical only	AP	+0.0006 (-0.0036 to +0.0029)	0.988	0.988
Angina	Primary incremental	Clinical + EC7 vs clinical only	Brier score	+0.00003 (-0.00002 to +0.00008)	0.221	0.221
Angina	Primary incremental	Clinical + EC7 vs clinical only	Brier skill score	-0.0006 (-0.0018 to +0.0005)	0.221	0.221
Angina	Primary incremental	Clinical + EC7 vs clinical only	Log loss	+0.00014 (-0.00015 to +0.00045)	0.322	0.322
Angina	Biomarker only	EC7 only vs established comparator proteins only	ROC-AUC	-0.1401 (-0.1700 to -0.1112)	<0.001	<0.001
Angina	Biomarker only	EC7 only vs established comparator proteins only	AP	-0.0363 (-0.0492 to -0.0248)	<0.001	<0.001
Angina	Biomarker only	EC7 only vs established comparator proteins only	Brier score	+0.00057 (+0.00020 to +0.00094)	0.002	0.004
Angina	Biomarker only	EC7 only vs established comparator proteins only	Brier skill score	-0.0127 (-0.0209 to -0.0045)	0.002	0.004
Angina	Biomarker only	EC7 only vs established comparator proteins only	Log loss	+0.00748 (+0.00497 to +0.00991)	<0.001	<0.001
Angina	Clinical biomarker	Clinical + EC7 vs clinical + established comparator	ROC-AUC	-0.0072 (-0.0137 to -0.0005)	0.036	0.036
Angina	Clinical biomarker	Clinical + EC7 vs clinical + established comparator	AP	-0.0050 (-0.0136 to +0.0030)	0.225	0.225
Angina	Clinical biomarker	Clinical + EC7 vs clinical + established comparator	Brier score	+0.00014 (-0.00007 to +0.00035)	0.231	0.231
Angina	Clinical biomarker	Clinical + EC7 vs clinical + established comparator	Brier skill score	-0.0031 (-0.0078 to +0.0016)	0.231	0.231
Angina	Clinical biomarker	Clinical + EC7 vs clinical + established comparator	Log loss	+0.00106 (+0.00002 to +0.00212)	0.046	0.046

Panel C. Additional descriptive paired comparisons

Outcome	Comparison family	Comparison (model A vs model B)	Metric	Difference (95% CI)	Bootstrap P value	FDR-adjusted q value
Stroke	Additional descriptive	Clinical + EC7 vs EC7 only	ROC-AUC	+0.1983 (+0.1605 to +0.2371)	<0.001	<0.001
Stroke	Additional descriptive	Clinical + EC7 vs EC7 only	AP	+0.0335 (+0.0237 to +0.0491)	<0.001	<0.001
Stroke	Additional descriptive	Clinical + EC7 vs EC7 only	Brier score	-0.00038 (-0.00050 to -0.00025)	<0.001	0.001
Stroke	Additional descriptive	Clinical + EC7 vs EC7 only	Brier skill score	+0.0176 (+0.0118 to +0.0234)	<0.001	0.001
Stroke	Additional descriptive	Clinical + EC7 vs EC7 only	Log loss	-0.00810 (-0.00990 to -0.00628)	<0.001	<0.001
Stroke	Additional descriptive	Clinical + established comparator proteins vs established comparator proteins only	ROC-AUC	+0.0320 (+0.0108 to +0.0525)	0.005	0.005
Stroke	Additional descriptive	Clinical + established comparator proteins vs established comparator proteins only	AP	+0.0087 (+0.0003 to +0.0185)	0.044	0.059
Stroke	Additional descriptive	Clinical + established comparator proteins vs established comparator proteins only	Brier score	-0.00024 (-0.00034 to -0.00012)	<0.001	0.001
Stroke	Additional descriptive	Clinical + established comparator proteins vs established comparator proteins only	Brier skill score	+0.0110 (+0.0058 to +0.0160)	<0.001	0.001
Stroke	Additional descriptive	Clinical + established comparator proteins vs established comparator proteins only	Log loss	-0.00343 (-0.00470 to -0.00214)	<0.001	<0.001
Heart failure	Additional descriptive	Clinical + EC7 vs EC7 only	ROC-AUC	+0.1859 (+0.1557 to +0.2151)	<0.001	<0.001
Heart failure	Additional descriptive	Clinical + EC7 vs EC7 only	AP	+0.0932 (+0.0718 to +0.1208)	<0.001	<0.001
Heart failure	Additional descriptive	Clinical + EC7 vs EC7 only	Brier score	-0.00248 (-0.00306 to -0.00195)	<0.001	0.001
Heart failure	Additional descriptive	Clinical + EC7 vs EC7 only	Brier skill score	+0.0622 (+0.0490 to +0.0769)	<0.001	0.001
Heart failure	Additional descriptive	Clinical + EC7 vs EC7 only	Log loss	-0.02297 (-0.02646 to -0.01956)	<0.001	<0.001
Heart failure	Additional descriptive	Clinical + established comparator proteins vs established comparator proteins only	ROC-AUC	+0.0229 (+0.0135 to +0.0323)	<0.001	0.002
Heart failure	Additional descriptive	Clinical + established comparator proteins vs established comparator proteins only	AP	+0.0333 (+0.0171 to +0.0504)	<0.001	0.002

Outcome	Comparison family	Comparison (model A vs model B)	Metric	Difference (95% CI)	Bootstrap P value	FDR-adjusted q value
Heart failure	Additional descriptive	Clinical + established comparator proteins vs established comparator proteins only	Brier score	-0.00107 (-0.00153 to -0.00060)	<0.001	0.001
Heart failure	Additional descriptive	Clinical + established comparator proteins vs established comparator proteins only	Brier skill score	+0.0267 (+0.0152 to +0.0385)	<0.001	0.001
Heart failure	Additional descriptive	Clinical + established comparator proteins vs established comparator proteins only	Log loss	-0.00579 (-0.00769 to -0.00391)	<0.001	<0.001
Acute myocardial infarction	Additional descriptive	Clinical + EC7 vs EC7 only	ROC-AUC	+0.1636 (+0.1223 to +0.2018)	<0.001	<0.001
Acute myocardial infarction	Additional descriptive	Clinical + EC7 vs EC7 only	AP	+0.0233 (+0.0130 to +0.0352)	<0.001	<0.001
Acute myocardial infarction	Additional descriptive	Clinical + EC7 vs EC7 only	Brier score	-0.00028 (-0.00046 to -0.00011)	0.002	0.002
Acute myocardial infarction	Additional descriptive	Clinical + EC7 vs EC7 only	Brier skill score	+0.0105 (+0.0042 to +0.0173)	0.002	0.002
Acute myocardial infarction	Additional descriptive	Clinical + EC7 vs EC7 only	Log loss	-0.00637 (-0.00847 to -0.00431)	<0.001	<0.001
Acute myocardial infarction	Additional descriptive	Clinical + established comparator proteins vs established comparator proteins only	ROC-AUC	+0.0337 (+0.0146 to +0.0536)	0.003	0.004
Acute myocardial infarction	Additional descriptive	Clinical + established comparator proteins vs established comparator proteins only	AP	+0.0058 (-0.0027 to +0.0152)	0.171	0.171
Acute myocardial infarction	Additional descriptive	Clinical + established comparator proteins vs established comparator proteins only	Brier score	-0.00017 (-0.00035 to 0.00000)	0.049	0.049
Acute myocardial infarction	Additional descriptive	Clinical + established comparator proteins vs established comparator proteins only	Brier skill score	+0.0065 (+0.0001 to +0.0132)	0.049	0.049
Acute myocardial infarction	Additional descriptive	Clinical + established comparator proteins vs established comparator proteins only	Log loss	-0.00324 (-0.00482 to -0.00171)	<0.001	<0.001
Angina	Additional descriptive	Clinical + EC7 vs EC7 only	ROC-AUC	+0.1898 (+0.1617 to +0.2172)	<0.001	<0.001
Angina	Additional descriptive	Clinical + EC7 vs EC7 only	AP	+0.0575 (+0.0433 to +0.0740)	<0.001	<0.001

Outcome	Comparison family	Comparison (model A vs model B)	Metric	Difference (95% CI)	Bootstrap P value	FDR-adjusted q value
Angina	Additional descriptive	Clinical + EC7 vs EC7 only	Brier score	-0.00129 (-0.00176 to -0.00082)	<0.001	0.001
Angina	Additional descriptive	Clinical + EC7 vs EC7 only	Brier skill score	+0.0287 (+0.0183 to +0.0392)	<0.001	0.001
Angina	Additional descriptive	Clinical + EC7 vs EC7 only	Log loss	-0.01422 (-0.01739 to -0.01098)	<0.001	<0.001
Angina	Additional descriptive	Clinical + established comparator proteins vs established comparator proteins only	ROC-AUC	+0.0569 (+0.0406 to +0.0733)	<0.001	0.002
Angina	Additional descriptive	Clinical + established comparator proteins vs established comparator proteins only	AP	+0.0262 (+0.0151 to +0.0384)	<0.001	0.002
Angina	Additional descriptive	Clinical + established comparator proteins vs established comparator proteins only	Brier score	-0.00086 (-0.00125 to -0.00047)	<0.001	0.001
Angina	Additional descriptive	Clinical + established comparator proteins vs established comparator proteins only	Brier skill score	+0.0191 (+0.0104 to +0.0278)	<0.001	0.001
Angina	Additional descriptive	Clinical + established comparator proteins vs established comparator proteins only	Log loss	-0.00781 (-0.01014 to -0.00555)	<0.001	<0.001

Note: All feature sets were evaluated in the same outcome-specific Common12 locked test sample. Confidence intervals were obtained from 2,000 bootstrap replicates where available; calibration intercept and slope are reported as point estimates. In Panels B and C, differences are calculated as model A minus model B: positive differences favor model A for ROC-AUC, average precision, and Brier skill score, whereas negative differences favor model A for Brier score and log loss. P values were obtained from paired bootstrap comparisons, and Benjamini–Hochberg false-discovery-rate correction was applied within each prespecified comparison family. No model-development decisions were made using locked test data.

Table S5. Single-protein logistic association estimates across three prespecified adjustment models

Panel A. Model 1: age- and sex-adjusted associations

Outcome	Protein	N	Cases, n (%)	OR per 1-SD higher protein level	Robust SE	P value
Stroke	NEXN	39,519	856 (2.17%)	0.985 (0.922 to 1.053)	0.034	0.659
Stroke	SLMAP	39,519	856 (2.17%)	1.087 (1.018 to 1.161)	0.034	0.013
Stroke	ITPR1	39,519	856 (2.17%)	1.080 (1.019 to 1.144)	0.029	0.009
Stroke	CACNB1	39,519	856 (2.17%)	1.018 (0.954 to 1.087)	0.033	0.587
Stroke	HRC	39,519	856 (2.17%)	1.073 (1.005 to 1.146)	0.034	0.035
Stroke	RYR1	39,519	856 (2.17%)	1.052 (0.983 to 1.125)	0.034	0.141
Stroke	CACNA1C	39,519	856 (2.17%)	1.053 (0.993 to 1.116)	0.030	0.085
Heart failure	NEXN	39,374	1,624 (4.12%)	1.020 (0.971 to 1.072)	0.025	0.427
Heart failure	SLMAP	39,374	1,624 (4.12%)	1.118 (1.066 to 1.172)	0.024	<0.001
Heart failure	ITPR1	39,374	1,624 (4.12%)	1.106 (1.060 to 1.154)	0.022	<0.001
Heart failure	CACNB1	39,374	1,624 (4.12%)	0.994 (0.944 to 1.046)	0.026	0.807
Heart failure	HRC	39,374	1,624 (4.12%)	1.233 (1.182 to 1.285)	0.021	<0.001
Heart failure	RYR1	39,374	1,624 (4.12%)	1.006 (0.954 to 1.060)	0.027	0.826
Heart failure	CACNA1C	39,374	1,624 (4.12%)	0.982 (0.931 to 1.036)	0.027	0.508
Acute myocardial infarction	NEXN	39,271	1,064 (2.71%)	1.031 (0.971 to 1.094)	0.030	0.317
Acute myocardial infarction	SLMAP	39,271	1,064 (2.71%)	1.108 (1.045 to 1.174)	0.030	<0.001
Acute myocardial infarction	ITPR1	39,271	1,064 (2.71%)	1.113 (1.058 to 1.172)	0.026	<0.001
Acute myocardial infarction	CACNB1	39,271	1,064 (2.71%)	1.041 (0.980 to 1.106)	0.031	0.193
Acute myocardial infarction	HRC	39,271	1,064 (2.71%)	1.060 (0.995 to 1.130)	0.032	0.073
Acute myocardial infarction	RYR1	39,271	1,064 (2.71%)	0.987 (0.922 to 1.055)	0.034	0.697
Acute myocardial infarction	CACNA1C	39,271	1,064 (2.71%)	0.999 (0.932 to 1.071)	0.035	0.969
Angina	NEXN	38,636	1,807 (4.68%)	1.013 (0.966 to 1.062)	0.024	0.593
Angina	SLMAP	38,636	1,807 (4.68%)	1.063 (1.013 to 1.115)	0.024	0.013
Angina	ITPR1	38,636	1,807 (4.68%)	1.061 (1.014 to 1.110)	0.023	0.011
Angina	CACNB1	38,636	1,807 (4.68%)	1.004 (0.958 to 1.053)	0.024	0.864
Angina	HRC	38,636	1,807 (4.68%)	0.970 (0.920 to 1.024)	0.027	0.273

Outcome	Protein	N	Cases, n (%)	OR per 1-SD higher protein level	Robust SE	P value
Angina	RYR1	38,636	1,807 (4.68%)	0.980 (0.929 to 1.034)	0.027	0.471
Angina	CACNA1C	38,636	1,807 (4.68%)	1.027 (0.978 to 1.077)	0.025	0.284

Panel B. Model 2: clinically adjusted primary associations

Outcome	Protein	N	Cases, n (%)	OR per 1-SD higher protein level	Robust SE	P value	FDR-adjusted
Stroke	NEXN	31,395	655 (2.09%)	0.985 (0.911 to 1.065)	0.040	0.704	0.897
Stroke	SLMAP	31,395	655 (2.09%)	1.019 (0.943 to 1.101)	0.040	0.640	0.897
Stroke	ITPR1	31,395	655 (2.09%)	1.059 (0.988 to 1.136)	0.036	0.106	0.373
Stroke	CACNB1	31,395	655 (2.09%)	1.002 (0.930 to 1.079)	0.038	0.961	0.961
Stroke	HRC	31,395	655 (2.09%)	1.093 (1.024 to 1.167)	0.033	0.008	0.055
Stroke	RYR1	31,395	655 (2.09%)	1.056 (0.977 to 1.141)	0.040	0.168	0.470
Stroke	CACNA1C	31,395	655 (2.09%)	1.076 (1.006 to 1.152)	0.035	0.033	0.153
Heart failure	NEXN	31,272	1,284 (4.11%)	1.021 (0.964 to 1.080)	0.029	0.480	0.840
Heart failure	SLMAP	31,272	1,284 (4.11%)	1.065 (1.008 to 1.126)	0.028	0.025	0.142
Heart failure	ITPR1	31,272	1,284 (4.11%)	1.088 (1.033 to 1.146)	0.026	0.002	0.022
Heart failure	CACNB1	31,272	1,284 (4.11%)	0.984 (0.929 to 1.043)	0.030	0.592	0.897
Heart failure	HRC	31,272	1,284 (4.11%)	1.246 (1.191 to 1.305)	0.023	<0.001	<0.001
Heart failure	RYR1	31,272	1,284 (4.11%)	1.007 (0.948 to 1.069)	0.031	0.825	0.935
Heart failure	CACNA1C	31,272	1,284 (4.11%)	0.969 (0.913 to 1.029)	0.031	0.311	0.786
Acute myocardial	NEXN	31,193	842 (2.70%)	1.015 (0.949 to 1.086)	0.034	0.660	0.897
Acute myocardial	SLMAP	31,193	842 (2.70%)	1.060 (0.991 to 1.134)	0.034	0.091	0.362
Acute myocardial	ITPR1	31,193	842 (2.70%)	1.047 (0.981 to 1.118)	0.034	0.167	0.470
Acute myocardial	CACNB1	31,193	842 (2.70%)	1.028 (0.958 to 1.103)	0.036	0.444	0.828
Acute myocardial	HRC	31,193	842 (2.70%)	1.091 (1.026 to 1.161)	0.032	0.006	0.052
Acute myocardial	RYR1	31,193	842 (2.70%)	1.004 (0.929 to 1.086)	0.040	0.914	0.948
Acute myocardial	CACNA1C	31,193	842 (2.70%)	0.963 (0.893 to 1.040)	0.039	0.337	0.786
Angina	NEXN	30,674	1,446 (4.71%)	0.994 (0.941 to 1.050)	0.028	0.835	0.935
Angina	SLMAP	30,674	1,446 (4.71%)	1.009 (0.955 to 1.066)	0.028	0.749	0.912
Angina	ITPR1	30,674	1,446 (4.71%)	1.024 (0.968 to 1.084)	0.029	0.400	0.800

Outcome	Protein	N	Cases, n (%)	OR per 1-SD higher protein level	Robust SE	P value	FDR-adjusted
Angina	CACNB1	30,674	1,446 (4.71%)	1.025 (0.971 to 1.081)	0.027	0.369	0.794
Angina	HRC	30,674	1,446 (4.71%)	1.004 (0.950 to 1.061)	0.028	0.900	0.948
Angina	RYR1	30,674	1,446 (4.71%)	0.988 (0.930 to 1.050)	0.031	0.691	0.897
Angina	CACNA1C	30,674	1,446 (4.71%)	1.019 (0.963 to 1.077)	0.029	0.519	0.855

Panel C. Model 3: clinically adjusted associations with additional comparator-protein adjustment

Outcome	Protein	N	Cases, n (%)	OR per 1-SD higher protein level	Robust SE	P value
Stroke	NEXN	29,131	607 (2.08%)	0.978 (0.900 to 1.062)	0.042	0.594
Stroke	SLMAP	29,131	607 (2.08%)	0.996 (0.916 to 1.083)	0.043	0.930
Stroke	ITPR1	29,131	607 (2.08%)	1.033 (0.955 to 1.116)	0.040	0.420
Stroke	CACNB1	29,131	607 (2.08%)	0.992 (0.917 to 1.074)	0.040	0.850
Stroke	HRC	29,131	607 (2.08%)	0.968 (0.891 to 1.053)	0.043	0.452
Stroke	RYR1	29,131	607 (2.08%)	1.038 (0.956 to 1.127)	0.042	0.374
Stroke	CACNA1C	29,131	607 (2.08%)	1.079 (1.005 to 1.158)	0.036	0.035
Heart failure	NEXN	29,017	1,198 (4.13%)	1.025 (0.964 to 1.091)	0.032	0.427
Heart failure	SLMAP	29,017	1,198 (4.13%)	1.036 (0.973 to 1.103)	0.032	0.268
Heart failure	ITPR1	29,017	1,198 (4.13%)	1.051 (0.990 to 1.116)	0.030	0.103
Heart failure	CACNB1	29,017	1,198 (4.13%)	0.968 (0.908 to 1.031)	0.032	0.311
Heart failure	HRC	29,017	1,198 (4.13%)	1.044 (0.978 to 1.114)	0.033	0.196
Heart failure	RYR1	29,017	1,198 (4.13%)	0.988 (0.921 to 1.060)	0.036	0.732
Heart failure	CACNA1C	29,017	1,198 (4.13%)	0.948 (0.887 to 1.013)	0.034	0.112
Acute myocardial infarction	NEXN	28,936	787 (2.72%)	1.004 (0.935 to 1.077)	0.036	0.913
Acute myocardial infarction	SLMAP	28,936	787 (2.72%)	1.031 (0.959 to 1.109)	0.037	0.411
Acute myocardial infarction	ITPR1	28,936	787 (2.72%)	1.010 (0.938 to 1.088)	0.038	0.791
Acute myocardial infarction	CACNB1	28,936	787 (2.72%)	1.014 (0.944 to 1.090)	0.037	0.704
Acute myocardial infarction	HRC	28,936	787 (2.72%)	1.020 (0.945 to 1.102)	0.039	0.606
Acute myocardial infarction	RYR1	28,936	787 (2.72%)	1.010 (0.931 to 1.095)	0.041	0.815
Acute myocardial infarction	CACNA1C	28,936	787 (2.72%)	0.969 (0.895 to 1.048)	0.040	0.426
Angina	NEXN	28,466	1,359 (4.77%)	0.985 (0.930 to 1.042)	0.029	0.592

Outcome	Protein	N	Cases, n (%)	OR per 1-SD higher protein level	Robust SE	P value
Angina	SLMAP	28,466	1,359 (4.77%)	0.981 (0.926 to 1.039)	0.030	0.516
Angina	ITPR1	28,466	1,359 (4.77%)	0.992 (0.934 to 1.054)	0.031	0.797
Angina	CACNB1	28,466	1,359 (4.77%)	1.017 (0.962 to 1.075)	0.028	0.544
Angina	HRC	28,466	1,359 (4.77%)	0.936 (0.877 to 0.999)	0.033	0.046
Angina	RYR1	28,466	1,359 (4.77%)	0.989 (0.931 to 1.051)	0.031	0.731
Angina	CACNA1C	28,466	1,359 (4.77%)	1.022 (0.965 to 1.082)	0.029	0.464

Note: Odds ratios are reported per 1-standard-deviation higher relative protein level, using the standard deviation calculated in the full corresponding outcome-specific EC7 cohort before covariate complete-case restriction. Model 1 adjusted for age and sex; Model 2 additionally included the prespecified clinical covariates and was the primary association model; Model 3 additionally included the five Comparator5 proteins and was fitted in the outcome-specific Common12 complete-case sample. HC3 robust standard errors were used. Benjamini–Hochberg false-discovery-rate correction was applied only to the 28 Model 2 tests; Model 1 and Model 3 P values were unadjusted.

Table S6. Outcome-specific EC7 first-principal-component construction and association results

Panel A. Outcome-specific EC7-PC1 construction summary

Outcome	Full EC7 N	Explained variance	Explained variance, %	Sum of oriented loadings
Stroke	39,519	2.018	28.83%	2.122
Heart failure	39,374	2.017	28.81%	2.119
Acute myocardial infarction	39,271	2.020	28.86%	2.123
Angina	38,636	2.020	28.86%	2.122

Panel B. Outcome-specific loadings of the seven EC7 proteins

Outcome	Protein	PC1 loading
Stroke	NEXN	0.6139
Stroke	SLMAP	0.6147
Stroke	ITPR1	0.4265
Stroke	CACNB1	0.1105
Stroke	HRC	0.1929
Stroke	RYR1	0.0657
Stroke	CACNA1C	0.0977
Heart failure	NEXN	0.6145
Heart failure	SLMAP	0.6150
Heart failure	ITPR1	0.4263
Heart failure	CACNB1	0.1095
Heart failure	HRC	0.1921
Heart failure	RYR1	0.0645
Heart failure	CACNA1C	0.0969
Acute myocardial infarction	NEXN	0.6137
Acute myocardial infarction	SLMAP	0.6145
Acute myocardial infarction	ITPR1	0.4269

Outcome	Protein	PC1 loading
Acute myocardial infarction	CACNB1	0.1096
Acute myocardial infarction	HRC	0.1935
Acute myocardial infarction	RYR1	0.0663
Acute myocardial infarction	CACNA1C	0.0982
Angina	NEXN	0.6141
Angina	SLMAP	0.6147
Angina	ITPR1	0.4263
Angina	CACNB1	0.1078
Angina	HRC	0.1935
Angina	RYR1	0.0668
Angina	CACNA1C	0.0987

Panel C. Clinically adjusted associations of the standardized EC7-PC1 score

Outcome	N	Cases, n (%)	OR per 1-SD higher EC7-PC1 score	Robust SE	P value	FDR-adjusted
Stroke	31,395	655 (2.09%)	1.042 (0.964 to 1.126)	0.040	0.302	0.403
Heart failure	31,272	1,284 (4.11%)	1.102 (1.043 to 1.165)	0.028	<0.001	0.002
Acute myocardial infarction	31,193	842 (2.70%)	1.060 (0.991 to 1.134)	0.034	0.091	0.181
Angina	30,674	1,446 (4.71%)	1.012 (0.957 to 1.069)	0.028	0.682	0.682

Note: For each outcome, the first principal component was derived separately and without outcome labels from the seven standardized EC7 protein levels. The component was oriented so that the sum of its loadings was positive and was standardized to 1 standard deviation in the full outcome-specific EC7 cohort before clinical complete-case restriction. The reported explained variance is the first-component eigenvalue, with its explained-variance percentage shown separately. Association models used the prespecified clinical covariates and HC3 robust standard errors; Benjamini–Hochberg false-discovery-rate correction was applied across the four principal-component–outcome tests.

Table S7. Two-year and five-year landmark sensitivity analyses of single-protein logistic associations

Panel A. Two-year landmark sensitivity analysis

Outcome	Protein	N	Cases, n (%)	OR per 1-SD higher protein level	Robust SE	P value	FDR-adjusted
Stroke	NEXN	31,151	605 (1.94%)	1.003 (0.926 to 1.088)	0.041	0.934	0.959
Stroke	SLMAP	31,151	605 (1.94%)	1.051 (0.971 to 1.138)	0.040	0.217	0.506
Stroke	ITPR1	31,151	605 (1.94%)	1.067 (0.993 to 1.146)	0.037	0.079	0.316
Stroke	CACNB1	31,151	605 (1.94%)	1.007 (0.933 to 1.088)	0.039	0.852	0.959
Stroke	HRC	31,151	605 (1.94%)	1.092 (1.019 to 1.170)	0.035	0.013	0.072
Stroke	RYR1	31,151	605 (1.94%)	1.063 (0.982 to 1.150)	0.040	0.132	0.462
Stroke	CACNA1C	31,151	605 (1.94%)	1.093 (1.022 to 1.169)	0.034	0.010	0.070
Heart failure	NEXN	31,012	1,194 (3.85%)	1.016 (0.958 to 1.077)	0.030	0.603	0.888
Heart failure	SLMAP	31,012	1,194 (3.85%)	1.061 (1.001 to 1.124)	0.029	0.045	0.211
Heart failure	ITPR1	31,012	1,194 (3.85%)	1.076 (1.019 to 1.135)	0.028	0.009	0.070
Heart failure	CACNB1	31,012	1,194 (3.85%)	0.974 (0.917 to 1.034)	0.031	0.386	0.698
Heart failure	HRC	31,012	1,194 (3.85%)	1.222 (1.165 to 1.281)	0.024	<0.001	<0.001
Heart failure	RYR1	31,012	1,194 (3.85%)	1.006 (0.945 to 1.072)	0.032	0.848	0.959
Heart failure	CACNA1C	31,012	1,194 (3.85%)	0.974 (0.916 to 1.036)	0.031	0.399	0.698
Acute myocardial	NEXN	30,923	762 (2.46%)	1.021 (0.951 to 1.095)	0.036	0.568	0.884
Acute myocardial	SLMAP	30,923	762 (2.46%)	1.048 (0.976 to 1.125)	0.036	0.195	0.506
Acute myocardial	ITPR1	30,923	762 (2.46%)	1.045 (0.974 to 1.121)	0.036	0.217	0.506
Acute myocardial	CACNB1	30,923	762 (2.46%)	1.041 (0.967 to 1.120)	0.038	0.288	0.621
Acute myocardial	HRC	30,923	762 (2.46%)	1.095 (1.027 to 1.167)	0.032	0.005	0.070
Acute myocardial	RYR1	30,923	762 (2.46%)	1.006 (0.926 to 1.093)	0.042	0.893	0.959
Acute myocardial	CACNA1C	30,923	762 (2.46%)	0.965 (0.891 to 1.046)	0.041	0.386	0.698
Angina	NEXN	30,299	1,243 (4.10%)	0.994 (0.938 to 1.053)	0.030	0.838	0.959
Angina	SLMAP	30,299	1,243 (4.10%)	0.996 (0.939 to 1.056)	0.030	0.885	0.959
Angina	ITPR1	30,299	1,243 (4.10%)	1.014 (0.954 to 1.079)	0.031	0.653	0.914

Outcome	Protein	N	Cases, n (%)	OR per 1-SD higher protein level	Robust SE	P value	FDR-adjusted
Angina	CACNB1	30,299	1,243 (4.10%)	1.039 (0.982 to 1.100)	0.029	0.186	0.506
Angina	HRC	30,299	1,243 (4.10%)	0.998 (0.940 to 1.060)	0.031	0.959	0.959
Angina	RYR1	30,299	1,243 (4.10%)	1.009 (0.947 to 1.074)	0.032	0.787	0.959
Angina	CACNA1C	30,299	1,243 (4.10%)	1.021 (0.964 to 1.082)	0.030	0.472	0.778

Panel B. Five-year landmark sensitivity analysis

Outcome	Protein	N	Cases, n (%)	OR per 1-SD higher protein level	Robust SE	P value	FDR-adjusted
Stroke	NEXN	30,620	499 (1.63%)	1.008 (0.921 to 1.103)	0.046	0.865	0.931
Stroke	SLMAP	30,620	499 (1.63%)	1.044 (0.956 to 1.140)	0.045	0.336	0.886
Stroke	ITPR1	30,620	499 (1.63%)	1.082 (1.002 to 1.169)	0.040	0.046	0.351
Stroke	CACNB1	30,620	499 (1.63%)	1.008 (0.926 to 1.098)	0.043	0.846	0.931
Stroke	HRC	30,620	499 (1.63%)	1.069 (0.985 to 1.159)	0.041	0.110	0.438
Stroke	RYR1	30,620	499 (1.63%)	1.062 (0.979 to 1.153)	0.042	0.147	0.514
Stroke	CACNA1C	30,620	499 (1.63%)	1.115 (1.038 to 1.197)	0.036	0.003	0.038
Heart failure	NEXN	30,465	1,025 (3.36%)	1.015 (0.953 to 1.081)	0.032	0.651	0.931
Heart failure	SLMAP	30,465	1,025 (3.36%)	1.054 (0.991 to 1.121)	0.032	0.097	0.438
Heart failure	ITPR1	30,465	1,025 (3.36%)	1.060 (1.000 to 1.123)	0.030	0.050	0.351
Heart failure	CACNB1	30,465	1,025 (3.36%)	0.971 (0.910 to 1.037)	0.033	0.380	0.886
Heart failure	HRC	30,465	1,025 (3.36%)	1.214 (1.154 to 1.277)	0.026	<0.001	<0.001
Heart failure	RYR1	30,465	1,025 (3.36%)	1.003 (0.939 to 1.071)	0.034	0.940	0.975
Heart failure	CACNA1C	30,465	1,025 (3.36%)	0.981 (0.918 to 1.048)	0.034	0.563	0.931
Acute myocardial	NEXN	30,365	616 (2.03%)	1.018 (0.941 to 1.101)	0.040	0.662	0.931
Acute myocardial	SLMAP	30,365	616 (2.03%)	1.028 (0.950 to 1.113)	0.040	0.488	0.931
Acute myocardial	ITPR1	30,365	616 (2.03%)	1.033 (0.955 to 1.116)	0.040	0.419	0.903
Acute myocardial	CACNB1	30,365	616 (2.03%)	0.978 (0.898 to 1.065)	0.043	0.605	0.931
Acute myocardial	HRC	30,365	616 (2.03%)	1.071 (0.995 to 1.153)	0.037	0.066	0.370
Acute myocardial	RYR1	30,365	616 (2.03%)	1.015 (0.929 to 1.109)	0.045	0.747	0.931

Outcome	Protein	N	Cases, n (%)	OR per 1-SD higher protein level	Robust SE	P value	FDR-adjusted
Acute myocardial	CACNA1C	30,365	616 (2.03%)	0.948 (0.865 to 1.038)	0.046	0.246	0.764
Angina	NEXN	29,566	906 (3.06%)	1.014 (0.949 to 1.085)	0.034	0.675	0.931
Angina	SLMAP	29,566	906 (3.06%)	1.008 (0.941 to 1.080)	0.035	0.819	0.931
Angina	ITPR1	29,566	906 (3.06%)	1.007 (0.937 to 1.081)	0.036	0.854	0.931
Angina	CACNB1	29,566	906 (3.06%)	1.032 (0.965 to 1.104)	0.034	0.350	0.886
Angina	HRC	29,566	906 (3.06%)	0.991 (0.925 to 1.062)	0.035	0.803	0.931
Angina	RYR1	29,566	906 (3.06%)	1.000 (0.934 to 1.071)	0.035	0.993	0.993
Angina	CACNA1C	29,566	906 (3.06%)	1.019 (0.951 to 1.093)	0.035	0.588	0.931

Note: At each landmark, participants with the corresponding outcome on or before the landmark and participants without follow-up extending beyond the landmark were excluded; cases were incident events occurring after the landmark. Odds ratios are reported per 1-standard-deviation higher relative protein level using the standard deviation from the full corresponding outcome-specific EC7 cohort. Models used the prespecified clinical covariates and HC3 robust standard errors. Benjamini–Hochberg false-discovery-rate correction was applied separately to the 28 two-year tests and the 28 five-year tests.

Table S8. Exploratory Cox associations and target-protein proportional-hazards diagnostics

Panel A. Exploratory clinical-adjusted Cox associations

Outcome	Protein	N	Events, n (%)	HR per 1-SD higher protein level	SE	P value	FDR-adjusted
Stroke	NEXN	31,395	655 (2.09%)	1.002 (0.928 to 1.082)	0.039	0.954	0.957
Stroke	SLMAP	31,395	655 (2.09%)	1.042 (0.965 to 1.125)	0.039	0.291	0.587
Stroke	ITPR1	31,395	655 (2.09%)	1.058 (0.982 to 1.139)	0.038	0.140	0.436
Stroke	CACNB1	31,395	655 (2.09%)	1.002 (0.929 to 1.081)	0.039	0.957	0.957
**Stroke**	**HRC**	31,395	655 (2.09%)	**1.108 (1.038 to 1.182)**	0.033	0.002	**0.015**
Stroke	RYR1	31,395	655 (2.09%)	1.056 (0.988 to 1.130)	0.034	0.110	0.386
Stroke	CACNA1C	31,395	655 (2.09%)	1.077 (1.004 to 1.154)	0.035	0.037	0.150
Heart failure	NEXN	31,272	1,284 (4.11%)	1.037 (0.982 to 1.095)	0.028	0.186	0.474
**Heart failure**	**SLMAP**	31,272	1,284 (4.11%)	**1.087 (1.030 to 1.147)**	0.028	0.002	**0.015**
**Heart failure**	**ITPR1**	31,272	1,284 (4.11%)	**1.083 (1.028 to 1.140)**	0.026	0.003	**0.015**
Heart failure	CACNB1	31,272	1,284 (4.11%)	0.988 (0.935 to 1.044)	0.028	0.663	0.813
**Heart failure**	**HRC**	31,272	1,284 (4.11%)	**1.224 (1.180 to 1.269)**	0.019	<0.001	**<0.001**
Heart failure	RYR1	31,272	1,284 (4.11%)	1.005 (0.952 to 1.061)	0.028	0.853	0.957
Heart failure	CACNA1C	31,272	1,284 (4.11%)	0.972 (0.918 to 1.029)	0.029	0.327	0.587
Acute myocardial infarction	NEXN	31,193	842 (2.70%)	1.029 (0.962 to 1.100)	0.034	0.402	0.587
Acute myocardial infarction	SLMAP	31,193	842 (2.70%)	1.083 (1.013 to 1.157)	0.034	0.020	0.091
Acute myocardial infarction	ITPR1	31,193	842 (2.70%)	1.049 (0.981 to 1.121)	0.034	0.161	0.449
Acute myocardial infarction	CACNB1	31,193	842 (2.70%)	1.028 (0.962 to 1.098)	0.034	0.414	0.587
**Acute myocardial infarction**	**HRC**	31,193	842 (2.70%)	**1.103 (1.040 to 1.170)**	0.030	0.001	**0.015**
Acute myocardial infarction	RYR1	31,193	842 (2.70%)	1.004 (0.938 to 1.075)	0.035	0.905	0.957
Acute myocardial infarction	CACNA1C	31,193	842 (2.70%)	0.967 (0.900 to 1.039)	0.037	0.362	0.587
Angina	NEXN	30,674	1,446 (4.71%)	1.002 (0.951 to 1.055)	0.026	0.950	0.957
Angina	SLMAP	30,674	1,446 (4.71%)	1.022 (0.970 to 1.076)	0.026	0.416	0.587

Outcome	Protein	N	Events, n (%)	HR per 1-SD higher protein level	SE	P value	FDR-adjusted
Angina	ITPR1	30,674	1,446 (4.71%)	1.026 (0.975 to 1.081)	0.026	0.321	0.587
Angina	CACNB1	30,674	1,446 (4.71%)	1.021 (0.971 to 1.075)	0.026	0.417	0.587
Angina	HRC	30,674	1,446 (4.71%)	1.015 (0.964 to 1.069)	0.026	0.565	0.753
Angina	RYR1	30,674	1,446 (4.71%)	0.988 (0.937 to 1.042)	0.027	0.668	0.813
Angina	CACNA1C	30,674	1,446 (4.71%)	1.022 (0.970 to 1.076)	0.026	0.419	0.587

Panel B. Target-protein proportional-hazards diagnostics

Outcome	Protein	PH slope	SE	Test statistic	P value	FDR-adjusted	PH diagnostic status
Stroke	NEXN	0.058	0.045	1.279	0.201	0.606	No flag
**Stroke**	**SLMAP**	0.114	0.045	2.534	**0.012**	**0.161**	**Nominal flag only**
Stroke	ITPR1	0.047	0.040	1.161	0.246	0.626	No flag
Stroke	CACNB1	0.008	0.042	0.187	0.851	0.954	No flag
Stroke	HRC	-0.018	0.035	-0.516	0.606	0.954	No flag
Stroke	RYR1	0.047	0.045	1.045	0.296	0.685	No flag
Stroke	CACNA1C	0.070	0.039	1.822	0.069	0.482	No flag
Heart failure	NEXN	-0.001	0.038	-0.014	0.989	0.989	No flag
Heart failure	SLMAP	-0.010	0.037	-0.282	0.778	0.954	No flag
Heart failure	ITPR1	-0.063	0.034	-1.834	0.067	0.482	No flag
Heart failure	CACNB1	-0.037	0.040	-0.934	0.350	0.685	No flag
**Heart failure**	**HRC**	-0.088	0.021	-4.081	**<0.001**	**0.001**	**FDR-significant flag**
Heart failure	RYR1	-0.015	0.041	-0.370	0.712	0.954	No flag
Heart failure	CACNA1C	0.011	0.040	0.272	0.786	0.954	No flag
Acute myocardial infarction	NEXN	0.008	0.039	0.217	0.828	0.954	No flag
Acute myocardial infarction	SLMAP	-0.056	0.039	-1.440	0.150	0.526	No flag
Acute myocardial infarction	ITPR1	-0.036	0.038	-0.950	0.342	0.685	No flag
Acute myocardial infarction	CACNB1	-0.002	0.040	-0.060	0.952	0.988	No flag
Acute myocardial infarction	HRC	-0.010	0.034	-0.309	0.757	0.954	No flag

Outcome	Protein	PH slope	SE	Test statistic	P value	FDR-adjusted	PH diagnostic status
Acute myocardial infarction	RYR1	0.041	0.046	0.902	0.367	0.685	No flag
Acute myocardial infarction	CACNA1C	-0.012	0.044	-0.268	0.788	0.954	No flag
Angina	NEXN	0.015	0.028	0.527	0.598	0.954	No flag
Angina	SLMAP	-0.035	0.028	-1.236	0.217	0.606	No flag
Angina	ITPR1	-0.045	0.029	-1.579	0.115	0.483	No flag
Angina	CACNB1	0.042	0.027	1.552	0.121	0.483	No flag
Angina	HRC	-0.003	0.028	-0.120	0.904	0.974	No flag
Angina	RYR1	0.049	0.032	1.554	0.121	0.483	No flag
Angina	CACNA1C	0.011	0.029	0.377	0.706	0.954	No flag

Note: Cox models were exploratory and used 31 October 2022 as the project-level proxy end date, derived from the maximum observed hospital diagnosis-record date available in the project extract. Hazard ratios are reported per 1-standard-deviation higher relative protein level using the standard deviation from the full corresponding outcome-specific EC7 cohort. Models used the prespecified clinical covariates, Efron handling of ties, and model-based standard errors. Benjamini–Hochberg false-discovery-rate correction was applied across the 28 association tests and separately across the 28 proportional-hazards diagnostics based on scaled Schoenfeld residuals. A nominal flag indicates $P<0.05$ without false-discovery-rate significance. The heart failure–HRC proportional-hazards diagnostic remained significant after correction; its constant summary hazard ratio should therefore be interpreted cautiously.

Table S9. Complete-case urinary albumin-to-creatinine ratio sensitivity analysis

Complete-case clinical and uACR-adjusted EC7 protein associations

Outcome	Protein	N	Cases, n (%)	Primary full-sample	Same-sample reduced OR	uACR-adjusted OR (95%	uACR-	uACR-adjusted	Change toward
Stroke	NEXN	9,729	287 (2.95%)	0.985 (0.911 to 1.065)	0.936 (0.833 to 1.052)	0.931 (0.828 to 1.047)	0.234	0.845	-7.8
Stroke	SLMAP	9,729	287 (2.95%)	1.019 (0.943 to 1.101)	0.918 (0.819 to 1.029)	0.907 (0.808 to 1.019)	0.100	0.698	-14.3
Stroke	ITPR1	9,729	287 (2.95%)	1.059 (0.988 to 1.136)	0.997 (0.889 to 1.118)	0.987 (0.879 to 1.109)	0.826	0.922	-301.3†
Stroke	CACNB1	9,729	287 (2.95%)	1.002 (0.930 to 1.079)	0.990 (0.878 to 1.117)	0.988 (0.876 to 1.115)	0.848	0.922	-20.4†
Stroke	HRC	9,729	287 (2.95%)	1.093 (1.024 to 1.167)	1.020 (0.919 to 1.132)	0.995 (0.897 to 1.104)	0.926	0.926	75.4
Stroke	RYR1	9,729	287 (2.95%)	1.056 (0.977 to 1.141)	1.017 (0.881 to 1.176)	1.017 (0.879 to 1.175)	0.824	0.922	5.2†
Stroke	CACNA1C	9,729	287 (2.95%)	1.076 (1.006 to 1.152)	1.136 (1.032 to 1.250)	1.137 (1.033 to 1.253)	0.009	0.123	-0.9
Heart failure	NEXN	9,668	605 (6.26%)	1.021 (0.964 to 1.080)	1.067 (0.984 to 1.158)	1.062 (0.978 to 1.154)	0.153	0.715	7.0
Heart failure	SLMAP	9,668	605 (6.26%)	1.065 (1.008 to 1.126)	1.067 (0.983 to 1.157)	1.051 (0.966 to 1.142)	0.247	0.845	23.4
Heart failure	ITPR1	9,668	605 (6.26%)	1.088 (1.033 to 1.146)	1.029 (0.948 to 1.116)	1.011 (0.930 to 1.100)	0.795	0.922	60.6
Heart failure	CACNB1	9,668	605 (6.26%)	0.984 (0.929 to 1.043)	1.025 (0.944 to 1.113)	1.022 (0.940 to 1.110)	0.614	0.922	12.7
**Heart failure**	**HRC**	**9,668**	**605 (6.26%)**	**1.246 (1.191 to 1.305)**	**1.253 (1.165 to 1.348)**	**1.213 (1.129 to 1.303)**	**<0.001**	**<0.001**	**14.6**
Heart failure	RYR1	9,668	605 (6.26%)	1.007 (0.948 to 1.069)	1.010 (0.927 to 1.100)	1.007 (0.923 to 1.099)	0.878	0.922	32.9†
Heart failure	CACNA1C	9,668	605 (6.26%)	0.969 (0.913 to 1.029)	0.959 (0.877 to 1.048)	0.959 (0.876 to 1.048)	0.355	0.845	-0.3
Acute myocardial	NEXN	9,670	327 (3.38%)	1.015 (0.949 to 1.086)	0.983 (0.886 to 1.092)	0.981 (0.884 to 1.090)	0.726	0.922	-12.5†
Acute myocardial	SLMAP	9,670	327 (3.38%)	1.060 (0.991 to 1.134)	1.044 (0.940 to 1.160)	1.038 (0.934 to 1.154)	0.486	0.922	12.9
Acute myocardial	ITPR1	9,670	327 (3.38%)	1.047 (0.981 to 1.118)	1.016 (0.914 to 1.130)	1.010 (0.908 to 1.125)	0.851	0.922	35.7†
Acute myocardial	CACNB1	9,670	327 (3.38%)	1.028 (0.958 to 1.103)	1.008 (0.898 to 1.132)	1.008 (0.898 to 1.132)	0.889	0.922	0.0†
Acute myocardial	HRC	9,670	327 (3.38%)	1.091 (1.026 to 1.161)	1.106 (1.001 to 1.222)	1.093 (0.987 to 1.209)	0.087	0.698	12.4
Acute myocardial	RYR1	9,670	327 (3.38%)	1.004 (0.929 to 1.086)	0.987 (0.874 to 1.115)	0.985 (0.872 to 1.113)	0.814	0.922	-14.5†
Acute myocardial	CACNA1C	9,670	327 (3.38%)	0.963 (0.893 to 1.040)	0.944 (0.832 to 1.070)	0.943 (0.831 to 1.069)	0.358	0.845	-1.7
Angina	NEXN	9,445	560 (5.93%)	0.994 (0.941 to 1.050)	1.039 (0.955 to 1.130)	1.038 (0.955 to 1.129)	0.380	0.845	1.7
Angina	SLMAP	9,445	560 (5.93%)	1.009 (0.955 to 1.066)	1.012 (0.929 to 1.104)	1.011 (0.927 to 1.102)	0.810	0.922	14.1†

Outcome	Protein	N	Cases, n (%)	Primary full-sample	Same-sample reduced OR	uACR-adjusted OR (95%	uACR-	uACR-adjusted	Change toward
Angina	ITPR1	9,445	560 (5.93%)	1.024 (0.968 to 1.084)	1.077 (0.982 to 1.181)	1.075 (0.980 to 1.180)	0.127	0.709	2.0
Angina	CACNB1	9,445	560 (5.93%)	1.025 (0.971 to 1.081)	1.043 (0.960 to 1.133)	1.042 (0.959 to 1.133)	0.326	0.845	0.6
Angina	HRC	9,445	560 (5.93%)	1.004 (0.950 to 1.061)	1.025 (0.939 to 1.118)	1.020 (0.933 to 1.115)	0.660	0.922	18.3
Angina	RYR1	9,445	560 (5.93%)	0.988 (0.930 to 1.050)	0.968 (0.872 to 1.075)	0.968 (0.871 to 1.074)	0.538	0.922	-1.5
Angina	CACNA1C	9,445	560 (5.93%)	1.019 (0.963 to 1.077)	0.962 (0.880 to 1.051)	0.962 (0.880 to 1.051)	0.392	0.845	0.6

Note: Primary full-sample Model 2 estimates are shown for context. The same-sample reduced and uACR-adjusted models used identical uACR-complete participants; the latter additionally included standardized natural-log-transformed uACR. uACR was calculated as urinary albumin divided by urinary creatinine and multiplied by 1,000 and was not imputed or winsorized. Protein odds ratios use the standard deviation from the full corresponding outcome-specific EC7 cohort, and HC3 robust standard errors were applied. Reported P and q values refer to the protein term in the uACR-adjusted model, with Benjamini–Hochberg correction across the 28 tests. Change toward the null was calculated as $100\times(|\beta\_reduced|-|\beta\_uACR\text{-}adjusted|)/|\beta\_reduced|$; † indicates an unstable percentage because $|\beta\_reduced|<0.02$. Percentage changes are descriptive and should not be interpreted as mediation or causality.

Table S10. Descriptive selection characteristics associated with EC7 and Common12 completeness

Panel A1. Baseline-characteristic balance and clinical-variable missingness: complete versus incomplete EC7 measurements

Variable	Level or scale	Complete-panel group	Incomplete-panel group	Absolute SMD	Missing in complete group, %	Missing in incomplete group, %	Missing-rate difference, percentage points
Age, years	Median [IQR]	58.000 [50.000 to 64.000]	58.000 [50.000 to 64.000]	0.014	0.00%	0.00%	+0.00
Sex	Female (max |SMD|)	21,479 (54.12%)	7,088 (53.28%)	0.017	0.00%	0.00%	+0.00
Body mass index, kg/m²	Median [IQR]	26.777 [24.163 to 29.904]	26.809 [24.242 to 29.980]	0.014	0.48%	0.52%	-0.04
Smoking status	Current (max |SMD|)	4,266 (10.75%)	1,333 (10.02%)	0.024	0.48%	0.47%	+0.01
Systolic blood pressure, mmHg	Median [IQR]	136.500 [124.500 to 149.500]	136.000 [124.500 to 149.000]	0.006	0.09%	0.09%	+0.00
Diastolic blood pressure, mmHg	Median [IQR]	82.000 [75.000 to 89.000]	81.500 [75.000 to 88.500]	0.020	0.09%	0.09%	+0.00
LDL cholesterol, mmol/L	Median [IQR]	3.488 [2.907 to 4.106]	3.480 [2.900 to 4.093]	0.013	5.22%	4.63%	+0.59
Hypertension	Missing category (max |SMD|)	20 (0.05%)	12 (0.09%)	0.015	0.05%	0.09%	-0.04
Diabetes mellitus	Missing category (max |SMD|)	17 (0.04%)	10 (0.08%)	0.013	0.04%	0.08%	-0.03
Cholesterol-lowering medication	No (max |SMD|)	31,824 (80.18%)	10,572 (79.46%)	0.018	1.33%	1.38%	-0.05
Townsend deprivation index	Median [IQR]	-2.040 [-3.600 to 0.770]	-2.090 [-3.660 to 0.730]	0.014	0.13%	0.11%	+0.03
Income deprivation score	Median [IQR]	0.080 [0.050 to 0.160]	0.080 [0.050 to 0.160]	0.007	14.42%	12.15%	+2.27
Ethnicity	White (max |SMD|)	36,902 (92.97%)	12,514 (94.06%)	0.044	0.50%	0.47%	+0.03
Employment group	Unemployed (max |SMD|)	738 (1.86%)	218 (1.64%)	0.017	1.20%	1.08%	+0.12
Alcohol-use status	Never (max |SMD|)	1,900 (4.79%)	606 (4.56%)	0.011	0.26%	0.29%	-0.04
Urinary albumin-to-creatinine ratio, mg/mmol	Median [IQR]; SMD on log1p scale	1.066 [0.665 to 2.171]	1.126 [0.675 to 2.386]	0.086	68.67%	68.08%	+0.60

Panel A2. Baseline-characteristic balance and clinical-variable missingness: complete versus incomplete Common12 measurements

Variable	Level or scale	Complete-panel group	Incomplete-panel group	Absolute SMD	Missing in complete group, %	Missing in incomplete group, %	Missing-rate difference, percentage points
Age, years	Median [IQR]	58.000 [50.000 to 64.000]	58.000 [50.000 to 64.000]	0.005	0.00%	0.00%	+0.00
Sex	Male (max |SMD|)	16,910 (45.86%)	7,518 (46.63%)	0.015	0.00%	0.00%	+0.00
Body mass index, kg/m²	Median [IQR]	26.776 [24.162 to 29.896]	26.818 [24.241 to 29.991]	0.019	0.49%	0.48%	+0.01
Smoking status	Current (max |SMD|)	3,978 (10.79%)	1,621 (10.05%)	0.024	0.50%	0.45%	+0.05
Systolic blood pressure, mmHg	Median [IQR]	136.500 [124.500 to 149.500]	136.000 [124.500 to 149.500]	0.001	0.09%	0.09%	+0.01
Diastolic blood pressure, mmHg	Median [IQR]	82.000 [75.000 to 89.000]	81.500 [75.000 to 88.500]	0.014	0.09%	0.09%	+0.01
LDL cholesterol, mmol/L	Median [IQR]	3.490 [2.910 to 4.108]	3.477 [2.896 to 4.091]	0.020	5.23%	4.71%	+0.52
Hypertension	No (max |SMD|)	25,779 (69.91%)	11,150 (69.16%)	0.016	0.05%	0.07%	-0.02
Diabetes mellitus	No (max |SMD|)	34,750 (94.24%)	15,156 (94.00%)	0.010	0.05%	0.06%	-0.02
Cholesterol-lowering medication	Yes (max |SMD|)	6,803 (18.45%)	3,084 (19.13%)	0.017	1.34%	1.35%	+0.00
Townsend deprivation index	Median [IQR]	-2.030 [-3.600 to 0.780]	-2.090 [-3.660 to 0.730]	0.016	0.13%	0.11%	+0.02
Income deprivation score	Median [IQR]	0.080 [0.050 to 0.160]	0.080 [0.050 to 0.160]	0.003	14.49%	12.39%	+2.10
Ethnicity	White (max |SMD|)	34,315 (93.07%)	15,101 (93.66%)	0.024	0.50%	0.48%	+0.02
Employment group	Unemployed (max |SMD|)	694 (1.88%)	262 (1.63%)	0.020	1.19%	1.13%	+0.06
Alcohol-use status	Never (max |SMD|)	1,766 (4.79%)	740 (4.59%)	0.009	0.26%	0.28%	-0.02
Urinary albumin-to-creatinine ratio, mg/mmol	Median [IQR]; SMD on log1p scale	1.064 [0.663 to 2.164]	1.121 [0.677 to 2.369]	0.087	68.68%	68.16%	+0.53

Panel B. Unadjusted incident outcome rates by protein-panel completeness

Completeness definition	Outcome	Complete group, N	Cases in complete group, n (%)	Incomplete group, N	Cases in incomplete group, n (%)	Event-rate difference, percentage points
EC7 completeness	Stroke	39,519	856 (2.17%)	13,238	290 (2.19%)	-0.02
EC7 completeness	Heart failure	39,374	1,624 (4.12%)	13,192	626 (4.75%)	-0.62
EC7 completeness	Acute myocardial infarction	39,271	1,064 (2.71%)	13,142	382 (2.91%)	-0.20
EC7 completeness	Angina	38,636	1,807 (4.68%)	12,870	649 (5.04%)	-0.37
Common 12-protein completeness	Stroke	36,713	799 (2.18%)	16,044	347 (2.16%)	+0.01
Common 12-protein completeness	Heart failure	36,576	1,510 (4.13%)	15,990	740 (4.63%)	-0.50
Common 12-protein completeness	Acute myocardial infarction	36,472	995 (2.73%)	15,941	451 (2.83%)	-0.10
Common 12-protein completeness	Angina	35,893	1,701 (4.74%)	15,613	755 (4.84%)	-0.10

Note: Panels A1 and A2 report continuous variables as median [interquartile range] and, for categorical variables, the category with the largest absolute standardized mean difference (SMD); missing values were retained as a category. uACR is displayed on its original mg/mmol scale, whereas its SMD was calculated after log1p transformation. Missing-rate differences were calculated as complete-panel minus incomplete-panel percentages. Panel B reports unadjusted outcome rates after outcome-specific eligibility and prevalent-disease exclusions; event-rate differences were calculated as complete-panel minus incomplete-panel rates. No null-hypothesis significance testing was performed, and these descriptive comparisons do not establish random missingness or statistical equivalence.

Table S11. EC7 prediction-algorithm screening and nested hyperparameter-tuning results

Panel A. Five-fold training-set algorithm screening: Stroke

Rank	Algorithm	Mean ROC-AUC (SD)	Mean AP (SD)	Balanced accuracy	Macro F1	Warnings, n	Advanced to nested
1	Logistic Regression	0.575 (0.013)	0.0316 (0.0047)	0.543	0.268	0	Yes
2	Shallow NN	0.560 (0.045)	0.0304 (0.0034)	0.534	0.337	0	Yes
3	AdaBoost	0.546 (0.024)	0.0249 (0.0017)	0.506	0.114	0	No
4	Gaussian Naive Bayes	0.546 (0.015)	0.0270 (0.0010)	0.524	0.281	0	No
5	Gradient Boosting	0.544 (0.032)	0.0269 (0.0029)	0.521	0.334	0	No
6	Random Forest	0.540 (0.032)	0.0254 (0.0017)	0.506	0.500	0	No
7	XGBoost	0.535 (0.038)	0.0260 (0.0034)	0.513	0.347	0	No
8	Bagging	0.529 (0.024)	0.0243 (0.0012)	0.505	0.494	0	No
9	MLP	0.525 (0.024)	0.0269 (0.0024)	0.523	0.443	0	No
10	KNN	0.521 (0.034)	0.0234 (0.0026)	0.516	0.454	0	No
11	LightGBM	0.513 (0.033)	0.0231 (0.0018)	0.515	0.441	10	No
12	SVM	0.512 (0.044)	0.0253 (0.0035)	0.506	0.365	5	No
13	Decision Tree	0.503 (0.012)	0.0219 (0.0006)	0.503	0.473	0	No

Panel B. Five-fold training-set algorithm screening: Heart failure

Rank	Algorithm	Mean ROC-AUC (SD)	Mean AP (SD)	Balanced accuracy	Macro F1	Warnings, n	Advanced to nested tuning
1	Logistic Regression	0.591 (0.029)	0.0664 (0.0057)	0.536	0.273	0	Yes
2	AdaBoost	0.584 (0.020)	0.0611 (0.0044)	0.522	0.153	0	Yes
3	Shallow NN	0.577 (0.030)	0.0645 (0.0080)	0.541	0.315	0	No
4	Gradient Boosting	0.569 (0.025)	0.0650 (0.0067)	0.533	0.319	0	No
5	Gaussian Naive Bayes	0.567 (0.037)	0.0609 (0.0062)	0.535	0.294	0	No
6	XGBoost	0.565 (0.021)	0.0634 (0.0064)	0.535	0.334	0	No
7	SVM	0.537 (0.018)	0.0507 (0.0032)	0.523	0.335	5	No

Rank	Algorithm	Mean ROC-AUC (SD)	Mean AP (SD)	Balanced accuracy	Macro F1	Warnings, n	Advanced to nested tuning
8	LightGBM	0.532 (0.019)	0.0555 (0.0049)	0.520	0.417	10	No
9	Random Forest	0.531 (0.023)	0.0514 (0.0041)	0.524	0.508	0	No
10	MLP	0.528 (0.028)	0.0502 (0.0064)	0.525	0.409	0	No
11	Bagging	0.527 (0.030)	0.0502 (0.0047)	0.521	0.500	0	No
12	KNN	0.513 (0.019)	0.0432 (0.0021)	0.513	0.444	0	No
13	Decision Tree	0.509 (0.018)	0.0422 (0.0017)	0.509	0.468	0	No

Panel C. Five-fold training-set algorithm screening: Acute myocardial infarction

Rank	Algorithm	Mean ROC-AUC (SD)	Mean AP (SD)	Balanced accuracy	Macro F1	Warnings, n	Advanced to nested tuning
1	Logistic Regression	0.563 (0.024)	0.0353 (0.0020)	0.538	0.255	0	Yes
2	AdaBoost	0.549 (0.040)	0.0308 (0.0032)	0.527	0.163	0	Yes
3	Shallow NN	0.545 (0.028)	0.0330 (0.0039)	0.538	0.321	0	No
4	Gaussian Naive Bayes	0.537 (0.033)	0.0324 (0.0029)	0.508	0.272	0	No
5	XGBoost	0.523 (0.032)	0.0306 (0.0039)	0.515	0.332	0	No
6	Random Forest	0.523 (0.033)	0.0306 (0.0041)	0.499	0.496	0	No
7	Bagging	0.523 (0.035)	0.0300 (0.0035)	0.496	0.490	0	No
8	Gradient Boosting	0.522 (0.031)	0.0305 (0.0038)	0.517	0.311	0	No
9	Decision Tree	0.516 (0.029)	0.0285 (0.0021)	0.516	0.473	0	No
10	LightGBM	0.515 (0.033)	0.0313 (0.0047)	0.502	0.424	10	No
11	SVM	0.512 (0.036)	0.0297 (0.0040)	0.507	0.351	5	No
12	MLP	0.510 (0.039)	0.0285 (0.0031)	0.511	0.422	0	No
13	KNN	0.506 (0.015)	0.0277 (0.0008)	0.505	0.448	0	No

Panel D. Five-fold training-set algorithm screening: Angina

Rank	Algorithm	Mean ROC-AUC (SD)	Mean AP (SD)	Balanced accuracy	Macro F1	Warnings, n	Advanced to nested tuning
1	Logistic Regression	0.546 (0.019)	0.0590 (0.0044)	0.509	0.148	0	Yes

Rank	Algorithm	Mean ROC-AUC (SD)	Mean AP (SD)	Balanced accuracy	Macro F1	Warnings, n	Advanced to nested tuning
2	Shallow NN	0.528 (0.027)	0.0543 (0.0039)	0.509	0.262	0	Yes
3	XGBoost	0.521 (0.006)	0.0516 (0.0033)	0.511	0.292	0	No
4	AdaBoost	0.520 (0.024)	0.0488 (0.0031)	0.507	0.079	0	No
5	Bagging	0.517 (0.010)	0.0494 (0.0027)	0.499	0.486	0	No
6	Gradient Boosting	0.516 (0.007)	0.0522 (0.0049)	0.507	0.267	0	No
7	Random Forest	0.513 (0.005)	0.0496 (0.0019)	0.505	0.496	0	No
8	Gaussian Naive Bayes	0.512 (0.016)	0.0507 (0.0049)	0.507	0.188	0	No
9	LightGBM	0.510 (0.006)	0.0506 (0.0034)	0.503	0.394	10	No
10	Decision Tree	0.510 (0.015)	0.0479 (0.0016)	0.510	0.466	0	No
11	MLP	0.507 (0.013)	0.0523 (0.0049)	0.500	0.380	0	No
12	SVM	0.506 (0.009)	0.0493 (0.0023)	0.499	0.301	5	No
13	KNN	0.495 (0.008)	0.0461 (0.0011)	0.495	0.433	0	No

Panel E. Nested cross-validation results for screened candidates

Outcome	Candidate algorithm	Selection status	Outer folds	Mean outer ROC-AUC (SD)	Pooled OOF ROC-AUC	Mean outer AP (SD)	Pooled OOF AP
**Stroke**	**Logistic Regression**	**Selected**	**5**	**0.575 (0.013)**	**0.574**	**0.0316 (0.0047)**	**0.0288**
Stroke	Shallow NN	Alternative	5	0.558 (0.030)	0.556	0.0297 (0.0035)	0.0277
**Heart failure**	**Logistic Regression**	**Selected**	**5**	**0.591 (0.029)**	**0.590**	**0.0664 (0.0057)**	**0.0643**
Heart failure	AdaBoost	Alternative	5	0.583 (0.024)	0.578	0.0646 (0.0066)	0.0617
**Acute myocardial**	**Logistic Regression**	**Selected**	**5**	**0.563 (0.025)**	**0.563**	**0.0351 (0.0023)**	**0.0341**
Acute myocardial	AdaBoost	Alternative	5	0.548 (0.040)	0.541	0.0312 (0.0033)	0.0310
**Angina**	**Logistic Regression**	**Selected**	**5**	**0.546 (0.019)**	**0.545**	**0.0590 (0.0044)**	**0.0572**
Angina	Shallow NN	Alternative	5	0.524 (0.025)	0.524	0.0517 (0.0042)	0.0504

Panel F. Prespecified model selection and final full-training parameters

Outcome	Selected model	Alternative candidate	Alternative - logistic ΔROC-AUC	Paired SE	Final CV ROC-AUC / AP	Full training N (cases)	Final hyperparameters
**Stroke**	**Logistic Regression**	**Shallow NN**	**-0.017**	**0.014**	**0.575 / 0.0316**	**27,663 (599)**	**C=0.1; l1_ratio=1 (pure L1 regularization); solver=liblinear; max_iter=5000**
**Heart failure**	**Logistic Regression**	**AdaBoost**	**-0.008**	**0.010**	**0.591 / 0.0664**	**27,561 (1,137)**	**C=0.1; l1_ratio=0 (pure L2 regularization); solver=liblinear; max_iter=5000**
**Acute myocardial infarction**	**Logistic Regression**	**AdaBoost**	**-0.015**	**0.010**	**0.564 / 0.0350**	**27,489 (745)**	**C=0.003; l1_ratio=1 (pure L1 regularization); solver=liblinear; max_iter=5000**
**Angina**	**Logistic Regression**	**Shallow NN**	**-0.022**	**0.007**	**0.546 / 0.0590**	**27,045 (1,265)**	**C=100; l1_ratio=0 (pure L2 regularization); solver=liblinear; max_iter=5000**

Note: Panels A–D report five-fold training-set screening of the 13 candidate algorithms for stroke, heart failure, acute myocardial infarction, and angina, respectively. Algorithms were ranked by mean ROC-AUC, and the two highest-ranked candidates for each outcome advanced to nested tuning; average precision was a secondary screening metric. Panel E reports five-outer-fold, three-inner-fold nested cross-validation, including pooled out-of-fold predictions. Panel F reports the prespecified parsimony-rule decision and final full-training hyperparameters. An alternative algorithm replaced logistic regression only when its paired mean outer-fold ROC-AUC advantage exceeded one standard error of the paired differences; this criterion was not met for any outcome. Median imputation and standard scaling were fitted within each training fold, and SMOTEENN was applied only to training folds. Warning counts represent repeated software warnings; no screening or nested-tuning model failed. All reported values are training-set model-development estimates rather than locked-test performance. The final logistic-regression models were implemented in scikit-learn 1.9.0 using the liblinear solver with a maximum of 5,000 iterations. Regularization type was specified through the l1_ratio parameter: l1_ratio=1 corresponds to pure L1 regularization, whereas l1_ratio=0 corresponds to pure L2 regularization. No intermediate elastic-net mixture was selected.

Abbreviations: AP, average precision; CV, cross-validation; KNN, k-nearest neighbors; MLP, multilayer perceptron; NN, neural network; OOF, out-of-fold; ROC-AUC, area under the receiver operating characteristic curve; SD, standard deviation; SE, standard error; SVM, support vector machine.

Table S12. Locked cohort partitioning, calibration, and classification-threshold specifications for EC7 prediction models

Panel A. Locked outcome-specific cohort partitions

Outcome	Cohort	Source N (cases)	Training N (cases)	Training event rate, %	Locked test N (cases)	Test event rate, %	Test fraction, %	Train-test overlap, n	Assignment provenance
Stroke	Primary EC7	39,519 (856)	27,663 (599)	2.165	11,856 (257)	2.168	30.00	0	Original locked partition
Stroke	Common 12-protein	36,713 (799)	25,727 (558)	2.169	10,986 (241)	2.194	29.92	0	Inherited from primary EC7
Heart failure	Primary EC7	39,374 (1,624)	27,561 (1,137)	4.125	11,813 (487)	4.123	30.00	0	Original locked partition
Heart failure	Common 12-protein	36,576 (1,510)	25,586 (1,053)	4.116	10,990 (457)	4.158	30.05	0	Inherited from primary EC7
Acute myocardial infarction	Primary EC7	39,271 (1,064)	27,489 (745)	2.710	11,782 (319)	2.708	30.00	0	Original locked partition
Acute myocardial infarction	Common 12-protein	36,472 (995)	25,537 (700)	2.741	10,935 (295)	2.698	29.98	0	Inherited from primary EC7
Angina	Primary EC7	38,636 (1,807)	27,045 (1,265)	4.677	11,591 (542)	4.676	30.00	0	Original locked partition
Angina	Common 12-protein	35,893 (1,701)	25,091 (1,192)	4.751	10,802 (509)	4.712	30.10	0	Inherited from primary EC7

Panel B. Frozen calibration and classification-threshold specifications

Outcome	Selected model	Calibration method	Calibration training N (cases)	Calibration-stage test rows used	Frozen threshold	Frozen before test opening	Locked test N (cases)	Test-set opening status	Test-development
Stroke	Logistic Regression	Sigmoid	27,663 (599)	0	0.022014	Yes	11,856 (257)	Final evaluation only	0
Heart failure	Logistic Regression	Sigmoid	27,561 (1,137)	0	0.043144	Yes	11,813 (487)	Final evaluation only	0

Outcome	Selected model	Calibration method	Calibration training N (cases)	Calibration-stage test rows used	Frozen threshold	Frozen before test opening	Locked test N (cases)	Test-set opening status	Test-development
Acute myocardial infarction	Logistic Regression	Isotonic	27,489 (745)	0	0.023360	Yes	11,782 (319)	Final evaluation only	0
Angina	Logistic Regression	Isotonic	27,045 (1,265)	0	0.044624	Yes	11,591 (542)	Final evaluation only	0

Note: Panel A reports the frozen outcome-specific training and locked-test partitions. Common12 participants retained the assignments inherited from their corresponding primary EC7 cohorts, with no participant redrawing or train–test overlap. Panel B reports the selected logistic-regression model, calibration method, and classification threshold fixed using training data before the locked test sets were opened; no test observations were used during calibration or threshold selection. Locked test sets were used only for final evaluation. These internal test sets do not constitute external or independent validation, and the frozen thresholds do not establish clinical utility.
